# A20 and ABIN-1 cooperate in balancing CBM complex-triggered NF-κB signaling in activated T cells

**DOI:** 10.1007/s00018-022-04154-z

**Published:** 2022-01-31

**Authors:** Hongli Yin, Ozge Karayel, Ying-Yin Chao, Thomas Seeholzer, Isabel Hamp, Oliver Plettenburg, Torben Gehring, Christina Zielinski, Matthias Mann, Daniel Krappmann

**Affiliations:** 1 Research Unit Cellular Signal Integration, Molecular Targets and Therapeutics Center, Helmholtz Zentrum München-German Research Center for Environmental Health, Ingolstaedter Landstr. 1, 85764 Neuherberg, Germany; 2 Department of Proteomics and Signal Transduction, Max-Planck Institute of Biochemistry, Martinsried, Germany; 3 Department of Infection Immunology, Leibniz Institute for Natural Product Research and Infection Biology, Hans-Knöll-Institute and Friedrich Schiller University Jena, Jena, Germany; 4 Central Institute for Translational Cancer Research (TranslaTUM), Technical University of Munich, Munich, Germany; 5 Institute for Medicinal Chemistry, Molecular Targets and Therapeutics Center, Helmholtz Zentrum München-German Research Center for Environmental Health, 30167 Hannover, Germany; 6 Centre of Biomolecular Drug Research (BMWZ), Institute of Organic Chemistry, Leibniz Universität Hannover, 30167 Hannover, Germany

**Keywords:** Immune activation, T cell signaling, Immune suppression, TNFAIP3, TNIP1, CARMA1

## Abstract

T cell activation initiates protective adaptive immunity, but counterbalancing mechanisms are critical to prevent overshooting responses and to maintain immune homeostasis. The CARD11-BCL10-MALT1 (CBM) complex bridges T cell receptor engagement to NF-κB signaling and MALT1 protease activation. Here, we show that ABIN-1 is modulating the suppressive function of A20 in T cells. Using quantitative mass spectrometry, we identified ABIN-1 as an interactor of the CBM signalosome in activated T cells. A20 and ABIN-1 counteract inducible activation of human primary CD4 and Jurkat T cells. While A20 overexpression is able to silence CBM complex-triggered NF-κB and MALT1 protease activation independent of ABIN-1, the negative regulatory function of ABIN-1 depends on A20. The suppressive function of A20 in T cells relies on ubiquitin binding through the C-terminal zinc finger (ZnF)4/7 motifs, but does not involve the deubiquitinating activity of the OTU domain. Our mechanistic studies reveal that the A20/ABIN-1 module is recruited to the CBM complex via A20 ZnF4/7 and that proteasomal degradation of A20 and ABIN-1 releases the CBM complex from the negative impact of both regulators. Ubiquitin binding to A20 ZnF4/7 promotes destructive K48-polyubiquitination to itself and to ABIN-1. Further, after prolonged T cell stimulation, ABIN-1 antagonizes MALT1-catalyzed cleavage of re-synthesized A20 and thereby diminishes sustained CBM complex signaling. Taken together, interdependent post-translational mechanisms are tightly controlling expression and activity of the A20/ABIN-1 silencing module and the cooperative action of both negative regulators is critical to balance CBM complex signaling and T cell activation.

**Supplementary Information:**

The online version contains supplementary material available at 10.1007/s00018-022-04154-z.

## Introduction

Antigen recognition by the TCR initiates an adaptive immune response. Assembly of the CARD11/CARMA1-BCL10-MALT1 (CBM) signalosome bridges TCR/CD28 co-stimulation to canonical NF-κB signaling pathways and activates the MALT1 protease to drive survival, expansion, differentiation and effector functions of activated T cells [1, 2]. The activity of the CBM complex is tightly balanced by positive and negative factors that foster immune protection after infections and prevent overshooting responses leading to autoimmunity and inflammation. Positive regulators recruited to the active CBM core complex include the E3 ligases TRAF6 and LUBAC, consisting of HOIP, HOIL-1 and SHARPIN. By catalyzing the ubiquitination of BCL10 and MALT1, these E3 ligases trigger IKK/NF-κB activation [3–6]. In contrast, the deubiquitinating enzymes (DUBs) CYLD and A20 (TNFAIP3) act as negative regulators of TCR signaling that limit NF-κB activation in an adaptive immune response [7].

A20 restricts autoimmunity and inflammation by acting as a pivotal negative regulator of many immune and inflammatory pathways [8]. A20 deficiency in CD8 T cells leads to sustained NF-κB activation, increased antigen-dependent IL-2 and IFNγ production, and improved anti-tumor responses [9]. In CD4 T cells, A20 ablation promoted proliferation after TCR engagement, but also stronger susceptibility to cell death [10]. In line, A20-deficient donor CD4 and CD8 T cells were less viable, but produced more IFNγ in a model of graft-versus-host disease thereby elevating systemic inflammation [11]. These results underscore that deletion of A20 in T cells does not cause spontaneous auto-activation, but enhances T cell effector responses. Thus, as an NF-κB-induced gene A20 acts in a negative feedback loop to limit activation and augment survival of T cells.

A20 restrains inflammation by counteracting TNFα and TLR-induced NF-κB signaling and cell death pathways [12–14]. Mechanistically, A20 serves a dual function as an ubiquitin-editing enzyme. The N-terminal OTU domain of A20 can hydrolyze K48-, K63- and K11-linked ubiquitin chains [14]. In addition, A20 binds M1- and K63-linked ubiquitin chains through its C-terminal ZnF4 and ZnF7 and facilitates K48-polyubiquitination and degradation of substrates [14–18]. In T cells, upon recruitment to the CBM complex after TCR/CD28 engagement, A20 is prone to MALT1 substrate cleavage and proteasomal degradation, which is thought to release the T cells from the negative regulatory effect of A20 [3, 19]. The A20 OTU domain is able to hydrolyze K63-linked ubiquitin chains conjugated to MALT1, but in how far DUB activity or ubiquitin binding of A20 contributes to suppression of T cell signaling remains elusive [3]. This is especially relevant, because A20 DUB activity is largely dispensable, but intact A20 ZnF4 and ZnF7 motifs are essential for counteracting pro-inflammatory NF-κB, cell death and inflammation [20–23].

Various co-factors are binding to the C-terminal ZnF region to modulate ubiquitin binding and E3 ligase activity of cellular A20 [8, 24]. Among these, A20-binding inhibitor of NF-κB-1 (ABIN-1; also termed TNIP1) binds to A20 via the ABIN-1 homology domain1 (AHD1) and recruits M1- and K63-linked ubiquitin chains through the UBAN (Ubiquitin binding in ABIN and NEMO) domain [25–29]. Like A20, ABIN-1 regulates innate and inflammatory NF-κB and cell death responses and recent data suggest that A20 and ABIN-1 act synergistic in preserving cell survival of epithelial cells [28, 30, 31]. Mice carrying homozygous UBAN-defective ABIN-1 D485N-mutant alleles suffer from autoimmunity resulting from augmented TLR signaling in myeloid cells and B cells, indicating that ubiquitin binding is critical for counteracting innate immune pathways [27]. However, TCR-triggered T cell proliferation and activation was largely unaffected by the ABIN-1 UBAN mutation. Thus, a possible A20-dependent or -independent role of ABIN-1 for T cell signaling has not been resolved.

Using immunoprecipitation coupled with quantitative mass spectrometry (MS), we identified ABIN-1 as a CBM complex interaction partner in activated Jurkat T cells. We demonstrate that ABIN-1 and A20 cooperate by forming a negative regulatory module in T cells, which binds to the CBM signalosome to limit NF-κB and MALT1 protease activation following TCR/CD28 stimulation. We show that interdependent post-translational processes tightly co-balance the expression levels of A20 and ABIN-1, revealing that the combined action of both factors tunes the strength and the length of TCR signaling.

## Materials and methods

### Cell culture, treatments, reagents and antibodies

Jurkat T cells were grown in RPMI-1640 medium supplemented with 10% fetal calf serum (FCS) and 100 units/ml penicillin plus 100 g/ml streptomycin (P/S). HEK293 and HEK293T cells were cultured in DMEM supplemented with 10% FCS and 100 U/ml P/S, and passaged when confluence exceeded 80%. Human CD4 T cells were grown in RPMI-1640 medium supplemented with 10% FCS (filtered), 100 U/ml P/S, 1% GlutaMax (100 ×), 1% Na-Pyruvate, 1% Non-essential amino acids, and 50 mM-Mercaptoethanol in the presence of IL-2. Cells were maintained at 37 °C in a water-saturated atmosphere supplemented with 5% CO2. Jurkat T cells were stimulated with 200 ng/ml Phorbol 12-myristate 13-acetate (PMA; Merck Millipore) and 300 ng/ml Ionomycin (Calbiochem) (P/I) or anti-CD3 (1 µg/ml, #555336)/anti-CD28 (3.3 µg/ml, #555725) antibody (CD3/CD28) in the presence of anti-mouse IgG1 (1.65 µg/ml, #553440) and IgG2a (1.65 µg/ml, #553387) (all BD Pharmingen). For stimulation of primary T cells, cells were transferred to anti-CD3 (0.5 µg/ml) pre-coated 96-well plates and stimulated in the presence of soluble anti-CD28 and anti-mouse IgG1/IgG2a as described above. Inhibitor treatment was done with 25 μM proteasome inhibitor MG132 (1 h pre-treatment; Calbiochem), 1 μM MALT1 inhibitor MLT-985 [32] (4 h pre-treatment), 75 μM MALT1 inhibitor Z-VRPR-FMK (Enzo Life Sciences) (3 h pre-treatment) and 2.5 μM cIAP1/2 inhibitor birinapant (10 min pre-treatment; BioCat) solved in dimethylsulphoxide (DMSO).

### Generation of knockout Jurkat and primary human CD4 T cells

Single-guide RNAs (sgRNAs) targeting ABIN-1 (5′-GAGCTCAGCCAGGGGGTCGA-3′, clone34; or 5′-TTATACCTGTGAGCTCAGCC-3′, clone33), A20 (5′-GAGGCAATTGCCGTCACCTG-3′), and HOIL-1 (combination of 5′-ATGGACGAGAAGACCAAGAA-3′ and 5′-TGTACCACGATCTGGCACTG-3′) were cloned into BbsI–linearized pX458 vector. Transfection of Jurkat T cells and generation of KO Jurkat clones by serial dilution has been described [33]. Loss of protein expression was analyzed by Western blotting and genomic alterations were analyzed by sequencing of genomic DNA. CARD11, BCL10, MALT1, HOIP and TRAF6 KO cells have previously been described [6, 34–37]. For experiments with human CD4 T cells, peripheral blood mononuclear cells (PBMC) from healthy donors were freshly isolated by density gradient sedimentation using Ficoll-Paque Plus (GE Healthcare). CD4^+^ T cells were isolated from PBMC by positive selection with CD4-specific microbeads (Miltenyi Biotec) and were stimulated with plate-bound anti-CD3 (2 μg/ml, clone TR66, Enzo Life Sciences) and anti-CD28 (2 μg/ml CD28.2; BD Biosciences) for 3 days. Genes were depleted using Alt-R CRISPR-Cas9 system (Integrated DNA Technologies, IDT). In brief, crRNA and tracrRNA (both from Integrated DNA Technologies, IDT) were mixed with 1:1 ratio and incubated with Cas9 protein (Integrated DNA Technologies, IDT) for 20 min at RT to form RNP complex. 1 × 10^6^ activated human CD4 T cells were electroporated and RNP complex were delivered into cells with Neon transfection system (Thermo Fisher Scientific) with the optimum program (1600 V, 10 ms pulse width, 3 pulses). Electroporated cells were then immediately incubated with RPMI-1640 medium supplemented with 10% FCS (filtered), 100 U/ml P/S, 1% GlutaMax (100 ×), 1% Na-Pyruvate, 1% Non-essential amino acids, and 50 mM-Mercaptoethanol in the presence of IL-2 (500 U/ml).

### Lentiviral transduction of Jurkat T cells and transfection of HEK293 cells

For stable expression of proteins in parental or KO Jurkat T cells, cells were transduced with lentiviruses expressing pHAGE-hΔCD2-T2A-A20-Flag-Strep-Strep or pHAGE-hΔCD2-T2A-ABIN-1-Flag-Strep-Strep WT or mutant (mt) constructs. Viral transduction has been performed as previously described [33]. For transient expression, Flag-A20, HA-A20, HA-ABIN-1, Flag-ABIN-1, HA-K48-Ub (K48-only), HA-K63-Ub (K63-only), and HA-WT Ub constructs were cloned into a pEF4 expression vector, and transfection of HEK293 cells was carried out using standard calcium phosphate transfection protocols.

### Expression of cytokines in primary T cells

For cytokine induction and analyses by flow cytometry and ELISA, primary human T cells were rested in T cell medium in absence of IL-2 for 24 h and stimulated with anti-CD3/CD28 as indicated above. For detection of intracellular cytokines by flow cytometry, stimulation was performed in presence of Brefeldin A (5 µg/ml, Sigma #B7651) for 5 h. Cells were washed with FACS buffer (PBS + 3% FCS) and stained for 30 min at 4 °C with Fixable Viability Dye eFluor 780 (1:1000 in FACS buffer, Invitrogen #65-0865-14). Cells were fixed in 2% PFA (15 min RT) and permeabilized in IC buffer (0.1% saponin in PBS) (15 min RT). Fc block (1:50 in IC buffer, eBioscience #14-9161-73) was performed for 7 min at RT. Cytokine staining was performed at 4 °C for 30 min in the dark using IL-2-APC (BD #554567) and TNFα-FITC (BD #554512) (both 1:100 in IC buffer). Cells were washed twice with IC buffer, resuspended in 100 μl FACS buffer and analyzed on an Attune Acoustic Focusing Cytometer. To determine secretion of IL-2, cells were stimulated for 24 h and analyzed using a human IL-2 ELISA kit according to the manufacturer’s instructions (Thermo Fisher Scientific #88-7025-88).

### Ubiquitination assay

To determine ubiquitination of endogenous MALT1, 3–5 × 10^7^ Jurkat cells were lysed in 450 μl co-IP buffer (25 mM HEPES pH 7.5, 150 mM NaCl, 0.2% NP40, 10% glycerol, 1 mM DTT, 10 mM NaF, 8 mM β-glycerophosphate, 300 µM sodium vanadate and protease inhibitor cocktail mix (Roche)) supplemented with 1% SDS. Lysates were homogenized through a syringe (26G) and boiled for 10 min before being lysed for 20 min at 4 °C, followed by three times successive centrifugation and transfer of supernatant (21,000×*g*, 20 min, 4 °C). Aliquots of supernatant were retained for Western blotting, the remaining supernatant was diluted tenfold with co-IP stock buffer without SDS or inhibitors, and subjected to immunoprecipitation. For detection of A20-triggered ABIN-1 ubiquitination or A20-triggered self-ubiquitination, HEK293 cells were transfected with Flag-A20 WT, OTU mt, ZnF4/7 mt, Flag-ABIN-1, HA-WT-Ub, HA-K63-Ub (K63-only) and HA-K48-Ub (K48-only) and lysed in 450 μl co-IP buffer containing 1% SDS supplemented with 10 mM *N*-ethylmaleimide (NEM; Thermo Fisher Scientific) and 50 μM MG132. Cells have been lysed as described above. For co-IP, overnight immunoprecipitation at 4 °C was performed with 250 μl MALT1 antibody (21A2, HMGU), 1 μg ABIN-1 antibody (#68-0002-100, Ubiquigent), 30 μl HA antibody (12CA5, HMGU) followed by 1–2 h incubation with 18 μl rec-protein G Sepharose 4B beads (1:1 suspension, Life Technologies). After this incubation, samples were centrifuged and the pellet was washed three times with co-IP buffer without protease and phosphatase inhibitors. The supernatant was removed, 25 μl 2 × SDS-PAGE loading buffer was added, beads were boiled at 95 °C for 5 min and analyzed by Western blot.

### Western blotting and electrophoretic mobility shift assays (EMSAs)

1–3 × 10^6^ cells were harvested (350×*g*, 5 min, 4 °C), washed with ice-cold PBS and resuspended in 100 µl high salt buffer (20 mM HEPES pH 7.9, 350 mM NaCl, 20% glycerol (v/v), 1 mM MgCl_2_, 0.5 mM EDTA, 0.1 mM EGTA, 1% NP40, 1 mM DTT, 10 mM NaF, 8 mM β-Glycerophosphate, 300 µM NaVanadate, and protease inhibitor cocktail (Roche)). Cells were lysed on a shaker for 20–30 min at 4 °C, and centrifuged for 30 min at 21,000×*g* at 4 °C. 20 μl of supernatant was removed and stored at − 80 °C for EMSA analysis. Western blot and EMSAs were performed as previously described [33]. Western blot images were recorded using the ECL Chemocam Imager (INTAS) with the ChemoStar Software (INTAS). Primary antibodies used for Western blot: ABIN-1 (Cat# 4664, RRID:AB_10547137), A20 (D13H3) (Cat# 5630, RRID:AB_10698880), CARD11 (1D12) (Cat# 4435, RRID:AB_10694496), p-IκBα (Cat# 9246, RRID:AB_2151442), IκBα (L35A5) (Cat# 4814, RRID: AB_390781) (all Cell Signaling Technology) β-Actin (C-4) (Cat# sc-47778, RRID:AB_626632), BCL10 (H-197) (Cat# sc-5611, RRID:AB_634292), CYLD (E-10) (Cat# sc-74435, RRID:AB_1122022), HOIL-1 (E-2) (Cat# sc-365523), MALT1 (B12) (Cat# sc-46677, RRID:AB_627909), Ubiquitin (P4D1, Cat# sc-8017) A20 (59A426, Cat# sc-52910, RRID:AB_771476) (all Santa Cruz Biotechnology), HOIP (Cat# MAB8039, RRID: AB_10676585), cIAP1 (Cat# AF8181, RRID: AB_2259001) (both R&D Systems). For secondary antibodies, we used HRP-conjugated anti-rabbit (Cat# 711-035-152, RRID: AB_10015282) and HRP-conjugated anti-mouse (Cat# 711-035-150, RRID: AB_2340770) (Jackson ImmunoResearch).

### Protein interaction studies

3–5 × 10^7^ cells were lysed in 800 μl co-IP buffer rotating for 20 min at 4 °C and centrifuged at 21,000×*g*, 15 min, 4 °C. Aliquots of supernatant were retained for Western blotting. For co-IP, overnight immunoprecipitation at 4 °C was performed with 0.5 μg BCL10 antibody (C-17, #sc9560), 1 μg ABIN-1 antibody (#68-0002-100, Ubiquigent) or 30 μl HA antibody (12CA5, HMGU), followed by 1–2 h incubation with 18 μl rec-protein G Sepharose 4B beads (1:1 suspension, Life Technologies). For Strep-PD (pull-down), cell lysates were incubated with 30 µl Strep-Tactin Sepharose (1:1 suspension, IBA) and rotated overnight at 4 °C. Beads were washed three times with ice-cold co-IP buffer without protease and phosphatase inhibitors. The supernatant was removed, 25 μl 2 × SDS-PAGE loading buffer was added, beads were boiled at 95 °C for 5 min and analyzed by Western blot.

### Flow cytometric analysis

To evaluate the hΔCD2 surface expression of infected cells, cells were stained with anti-CD2-APC antibody (1:400, Invitrogen #17-0029-42) at 4 °C in the dark for 20 min. To evaluate the NF-κB reporter gene activity of the EGFP reporter gene, cells were stimulated as described above, washed and resuspended in PBS, and analyzed on Attune Acoustic Focusing Cytometer. Data were analyzed using FlowJo software 10.7.1.

### Detection of active MALT1

Generation and application of biotin-labeled MALT1 activity-based probes (MALT1 ABPs) to precipitate and detect protease active MALT1 has been described previously [38]. MALT1 substrate cleavage was detected by Western Blot and for quantification of CYLD and HOIL-1 cleavage Fiji/ImageJ was used to measure band intensity of the cleaved and full-length MALT1 substrates and to calculate the ratios of cleaved to full-length substrates.

### Liquid chromatography and tandem mass spectrometry (LC–MS/MS)

For identification of BCL10 interaction partners, CK1α KO Jurkat T cells reconstituted with CK1α WT or D136N were used. 6 × 10^7^ cells per sample were left untreated or stimulated for 20 min with P/I before lysis in 1% NP40 buffer (150 mM NaCl, 50 mM Tris–HCl (pH 7,5), 10 mM Na-Pyrophosphate, 10 mM Na-Glycerophosphate, 1% NP40, 20 mM NaF, 1 mM EGTA, 1 mM EDTA, 1 mM DTT, 10% Glycerol and protease inhibitors). BCL10 IP (0.5 µg/sample anti-BCL10, C-17, sc-9560) was performed overnight and binding to Protein G Sepharose 4B beads (Life Technologies) was performed for 2 h at 4 °C. After IP, beads were washed 2 × in 1% NP40 buffer and 2 × in 50 mM Tris–HCl (pH 7.5) buffer. On-bead digestion was performed overnight at 37 °C after re-suspending the beads in 2 M urea dissolved in 50 mM Tris–HCl (pH 7.5) buffer and adding trypsin. The generated peptides were cleaned using in-house prepared SDB-RPS (Empore) Stage Tips [39] prior to LC–MS/MS analysis as described previously [40].

### RNA extraction and quantitative reverse-transcriptase polymerase chain reaction (qRT-PCR)

Total RNA was isolated with RNeasy Kit (QIAGEN) and transcribed into cDNA using the Verso cDNA synthesis Kit (Thermo Fisher Scientific) according to the manufacturer’s guide. Primer sequences of the *TNIP1*, *TNFAIP3* and *RPII* genes are as follows (forward and reverse, respectively): *ABIN-1/TNIP1*, 5′-GTTCAACCGACTGGCATCCAA-3′ and 5′-AGACGCACCCTCTTTGTTGC-3′; *A20/TNFAIP3*, 5′-CTGAAAACGAACGGTGACGG-3′ and 5′-CGTGTGTCTGTTTCCTTGAGCG-3′; *RPII*, 5′-GTTCGGAGTCCTGAGTCCGGATG-3′ and 5′-CCTGCCTCGGGTCCATCAGC-3′. For quantitative real-time PCR, the LightCycler 480 instrument (Roche) and Takyon qPCR Kit for SYBR Assays (Eurogentec) was used with a standard LightCycler protocol. RNA polymerase II (*RPII*) was used as an internal standard.

### Statistics summary

Statistical analyses and data visualization were performed in GraphPad Prism version 8.0. Statistical details can be found in the figure legends, including the statistical tests used for calculating *p* values.

## Results

### Identification of the CBM signalosome by mass spectrometry in Jurkat T cells

**Fig. 1:**
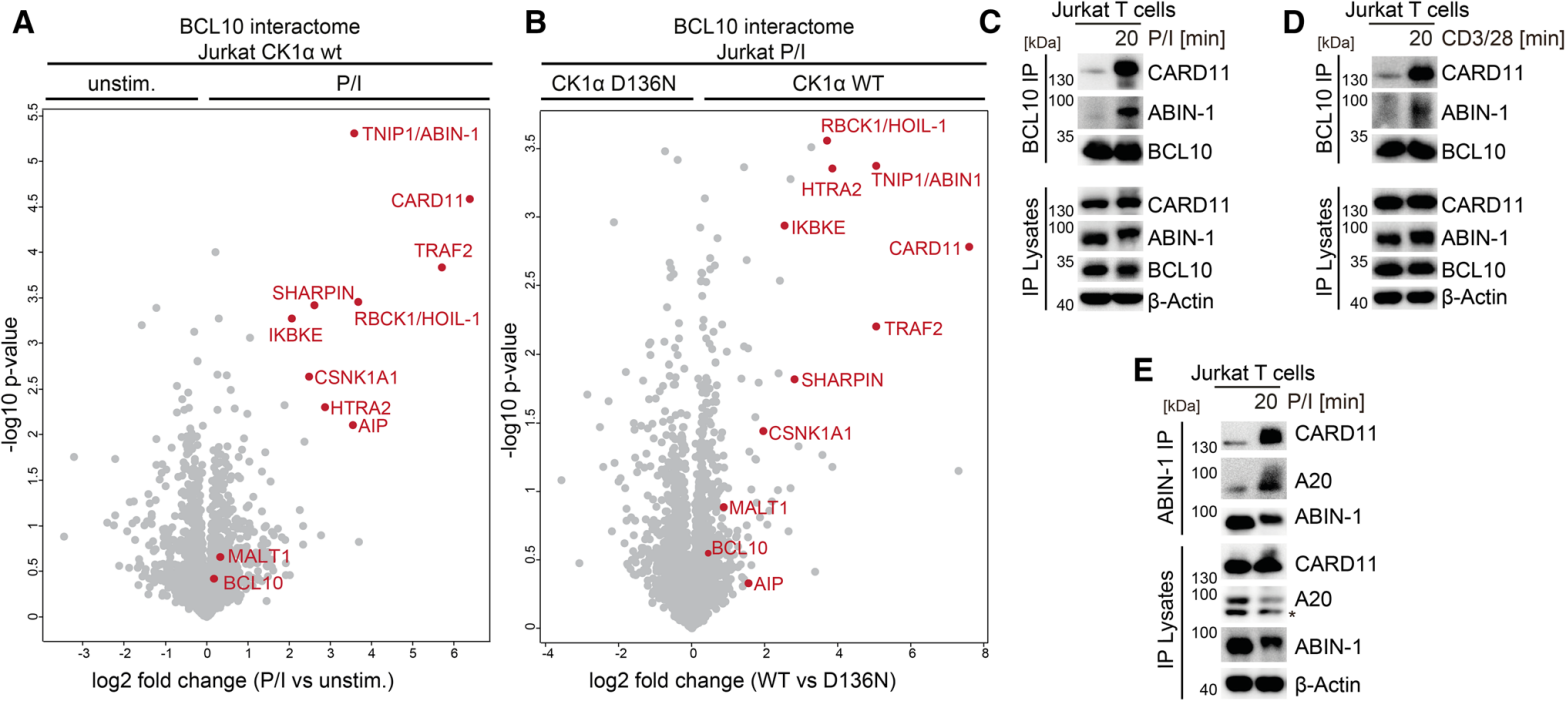
ABIN-1 interacts with the CBM complex following T cell stimulation. **A** Enrichment of proteins interacting with BCL10 after IP comparing unstimulated and P/I stimulated (20 min) CK1α WT Jurkat T cells. Volcano plot illustrates the (− log10) *p* values with respect to the log2 abundance differences of proteins identified in BCL10 IPs between P/I stimulated versus unstimulated (*n* = 3). **B** Enrichment of proteins interacting with BCL10 after IP comparing CK1α WT and D136N Jurkat T cells after P/I stimulation (20 min). Volcano plot illustrates the (− log10) *p* values with respect to the log2 abundance differences of proteins identified in BCL10 IPs between CK1α WT versus D136N (*n* = 3). **C**, **D** BCL10 IP was performed in untreated or P/I or CD3/CD28 stimulated Jurkat T cells and binding of CARD11 and ABIN-1 was analyzed by Western blot. **E** ABIN-1 IP was performed in untreated or P/I stimulated Jurkat T cells and binding of CARD11 and A20 was analyzed by Western blot

Casein kinase 1α (CK1α) activity is essential for CBM complex assembly in activated T cells [33]. Thus, we used Jurkat T cells expressing either CK1α WT or kinase-dead D136N mutant to identify CK1α activity-dependent recruitment of co-factors to the CBM signalosome. We employed LC/MS–MS after BCL10 IP, which efficiently enriches the inducible CBM signaling complex after T cell stimulation with P/I [35] (Table S1). While MALT1 constitutively bound to BCL10 even in unstimulated T cells, CARD11 association and thus CBM complex formation required P/I stimulation (Fig. 1A). CARD11-BCL10 interaction strictly relied on CK1α kinase activity, confirming the critical role of CK1α for CBM signalosome assembly (Fig. 1B). With HOIL-1 (RBCK1), SHARPIN, CK1α (CSNK1A1), TRAF2 and AIP, we identified a number of known CBM complex interactors in activated T cells [41–45]. With the exception of AIP, binding of all co-factors identified was dependent on CK1α kinase activity (Fig. 1B). Further, we identified ABIN-1/TNIP1 and IKKε (IKBKE) as putative CBM complex interactors in activated T cells, but specific roles in for these proteins CBM complex-dependent T cell signaling have not been described.

We focused on ABIN-1, because its binding to BCL10 was highly enriched upon stimulation and we could confirm inducible binding after anti-BCL10 IP and Western blotting following P/I and TCR/CD28 stimulation of Jurkat T cells (Fig. 1C, D). ABIN-1 facilitates the negative regulatory impact of A20 on pro-inflammatory or innate immune signaling [46, 47], but its role in T cell signaling has not yet been described. Of note, despite the fact that A20 is a MALT1 substrate that counteracts CBM complex signaling, we did not detect A20 as part of the BCL10 interactome in resting or activated Jurkat T cells [3, 19] (Table S1). Thus, we performed ABIN-1-IP and confirmed that CARD11 and A20 associated with ABIN-1 in activated Jurkat T cells, suggesting a combined recruitment of ABIN-1 and A20 to the CBM complex (Fig. 1E).

### ABIN-1 and A20 antagonize activation of human primary T cells

**Fig. 2:**
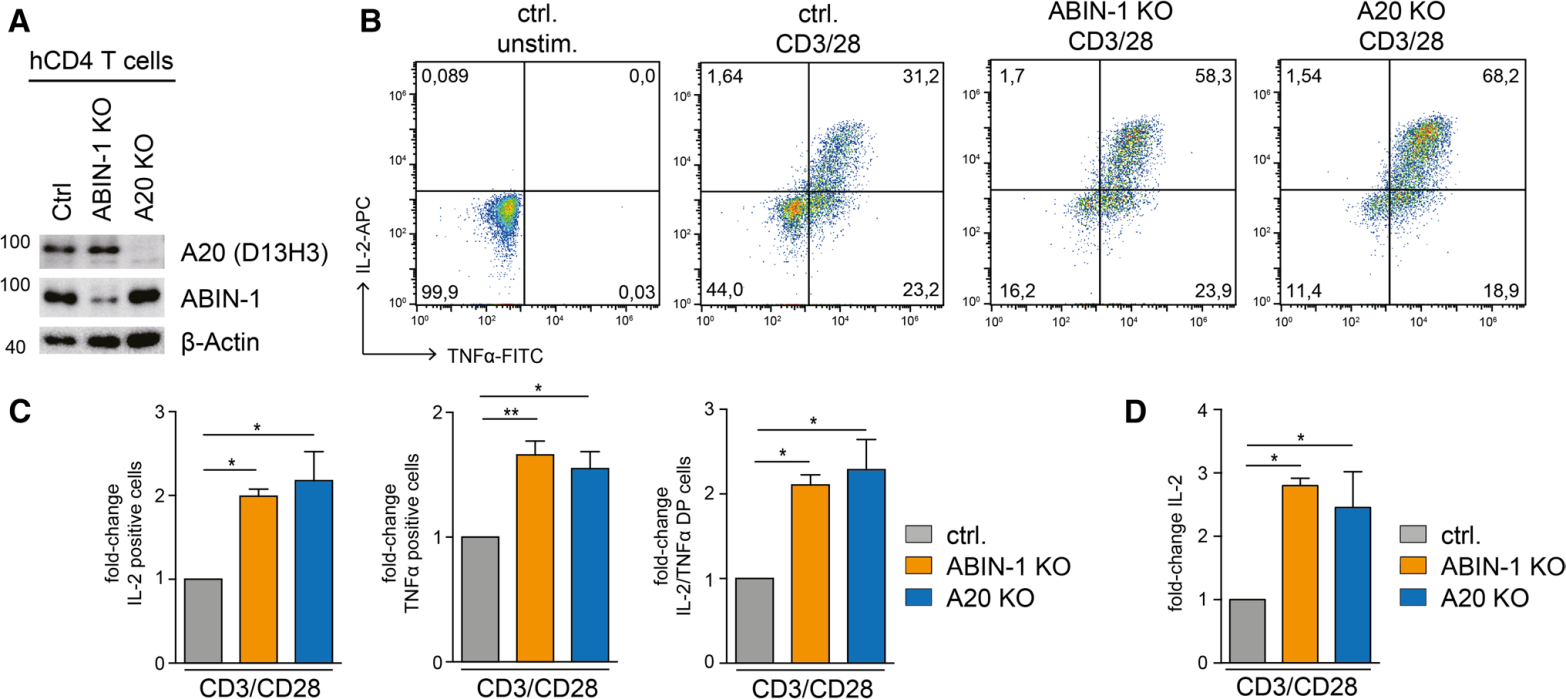
ABIN-1 and A20 deficiency increase effector response in primary human CD4 T cells. **A** ABIN-1 and A20 expression after CRISPR/Cas9 KO in CD4 T cells analyzed by Western blot. **B** Intracellular staining of IL-2 and TNFα production following CD3/CD28 stimulation (4 h) in ABIN-1 and A20 KO CD4 T cells. **C** Quantification of fold change IL-2, TNFα and IL-2/TNFα-positive cells as shown in B from three independent experiments. **D** Quantification of fold change of IL-2 secretion determined by ELISA in supernatant of ABIN-1 and A20 KO CD4 T cells stimulated for 18 h with CD3/CD28 from three independent experiments. Data represent means ± SEM (*n* = 3) and quantification was done by one-way ANOVA with Dunnett’s multiple comparisons test, shown above comparisons. *p < 0.05, **p < 0.01

To gain insights into the functional role of ABIN-1 and A20 in the regulation of T cell activation, we designed single guide (sg)RNAs targeting ABIN-1 or A20 by CRISPR/Cas9 technology. Transfection of ABIN-1 and A20 sgRNAs caused efficient ablation of ABIN-1 and A20 proteins in the pools of primary human CD4 T cells, respectively (Fig. 2A). We monitored induction of cytokines after T cell co-stimulation. On single cell level, the frequency of IL-2 and TNFα producing CD4 T cells was increased in ABIN-1 or A20 KO, which also promoted increased extracellular secretion of IL-2 in response to TCR/CD28 stimulation, demonstrating that both proteins are counteracting effector responses in primary human CD4 T cells (Fig. 2B–D).

### ABIN-1 and A20 counteract TCR-induced NF-κB and MALT1 protease activation

**Fig. 3:**
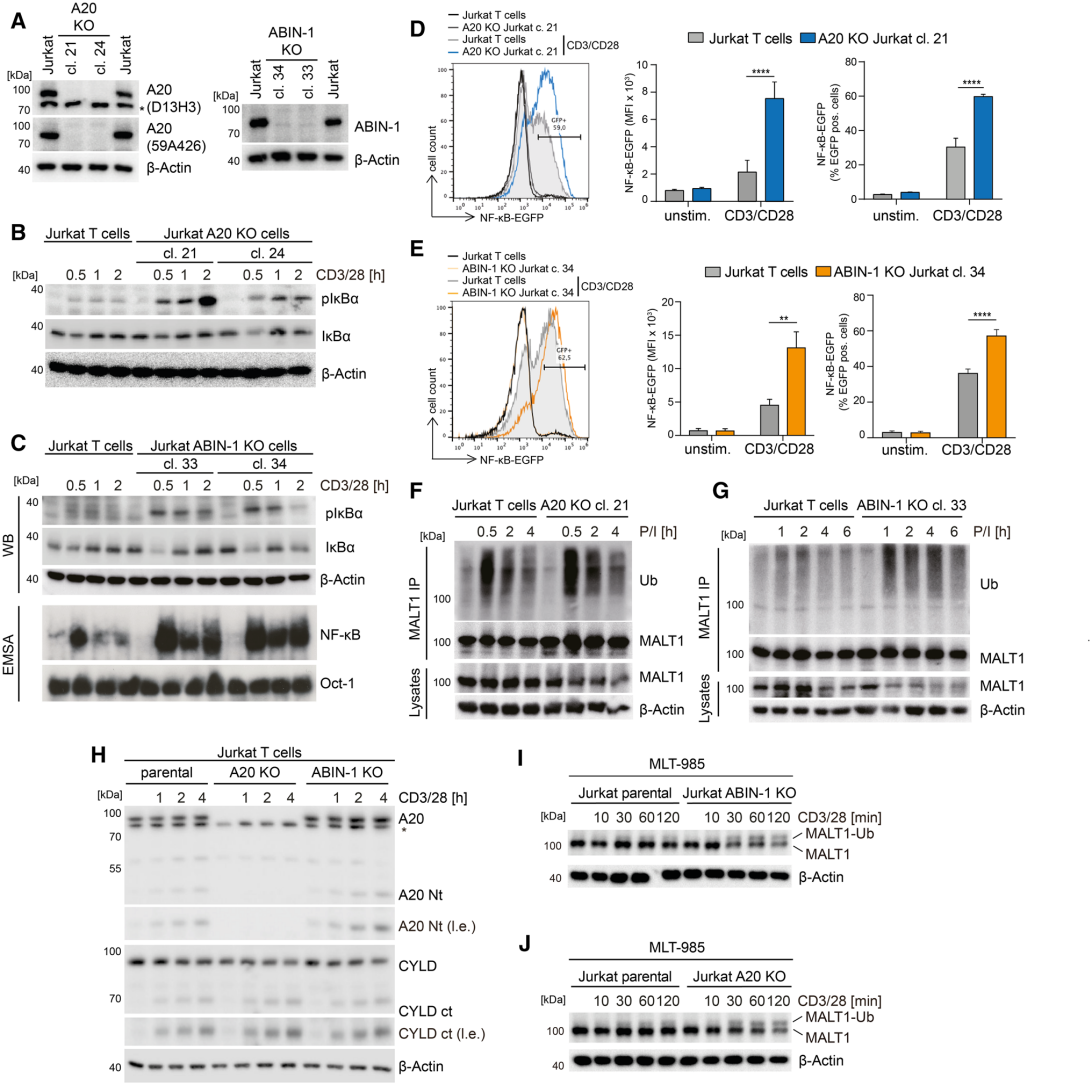
ABIN-1 and A20 deficiency augments CBM signaling in Jurkat T cells. **A** ABIN-1 and A20 expression after CRISPR/Cas9 KO in Jurkat T cells analyzed by Western blot. **B**, **C** NF-κB signaling in parental and KO Jurkat T cells following CD3/CD28 stimulation analyzed by Western blot and EMSA. **D**, **E** NF-κB-EGFP reporter activation in parental and KO Jurkat T cells following CD3/CD28 stimulation (4 h) analyzed by flow cytometry. Quantification was done by calculating median fluorescence intensity (MFI) and number of EGFP-positive cells. Data represent means ± SEM (*n* = 3) (**D**) or (n = 4) (**E**) and quantification was done by two-way ANOVA with Tukey’s multiple comparisons test, shown above comparisons. **p < 0.01, ****p < 0.0001. **F**, **G** MALT1 polyubiquitination following P/I stimulation. **H** Cleavage of MALT1 substrates was detected after CD3/CD28 stimulation by Western blot. **I**, **J** MALT1 mono-ubiquitination was detected by Western blot after MALT1 inhibition with MLT-985

To investigate and compare the molecular effects of A20 and ABIN-1 on T cell signaling, we generated KO Jurkat T cell clones using the same targeting strategy as in primary T cells (Fig. 3A). A20 or ABIN-1 KO Jurkat T cells displayed a robust increase in NF-κB signaling, as evident from augmented and prolonged IκBα phosphorylation and degradation after TCR/CD28 or P/I stimulation (Fig. 3B, C; Fig. S1A, B). As previously shown after A20 knockdown, NF-κB DNA-binding activity was also increased in ABIN-1 KO Jurkat T cells after TCR/CD28 stimulation (Fig. 3C) [3]. To analyze the NF-κB transcriptional response, we transduced a NF-κB-EGFP reporter into parental Jurkat T cells as well as A20 (clone 21) and ABIN-1 (clone 34) KO Jurkat T cells. After 4 h of TCR/CD28 co-stimulation, the median fluorescence intensity (MFI) as well as the percentage of EGFP-positive cells was increased in Jurkat T cells lacking A20 or ABIN-1, demonstrating strongly augmented NF-κB activation (Fig. 3D, E). NF-κB reporter activation after P/I stimulation was enhanced in A20 and ABIN-1 KO Jurkat T cells, but effects were less pronounced, because of the much stronger reporter gene induction in response to P/I (Fig. S1C and D). MALT1 polyubiquitination is suggested to trigger NF-κB activation by facilitating the recruitment of the IKK complex to the CBM complex upon T cell activation [3, 4]. Indeed, both A20 and ABIN-1 deficiency led to more robust polyubiquitination of MALT1 after P/I stimulation (Fig. 3F, G; Fig. S1E). The data demonstrate that ABIN-1 and A20 counteract CBM-mediated NF-κB activation in T cells in a highly similar manner, indicative for a cooperative mode of action.

Next, we assessed MALT1-catalyzed substrate cleavage in the absence of A20 and ABIN-1 in Jurkat T cells. A20 cleavage was enhanced in ABIN-1 KO cells after TCR/CD28 or P/I stimulation (Fig. 3H; Fig. S1F). There was a mild increase in the appearance of the CYLD cleavage product in A20 or ABIN-1 KO Jurkat T cells after CD3/CD28 stimulation, which was not visible after stronger P/I stimulation. MALT1 mono-ubiquitination serves as a critical signal for MALT1 protease activation [48]. Indeed, MALT1 mono-ubiquitination was enhanced in ABIN-1 and A20 KO Jurkat T cells stimulated with CD3/CD28 and treated with MALT1 inhibitor MLT-985 to enrich the active mono-ubiquitinated form of MALT1 (Fig. 3I, J) [49]. Thus, presence of ABIN-1 and A20 mildly impairs MALT1 mono-ubiquitination and protease activation in Jurkat T cell.

### ABIN-1 counteracts TCR-induced NF-κB signaling and MALT1 protease activation via A20

**Fig. 4:**
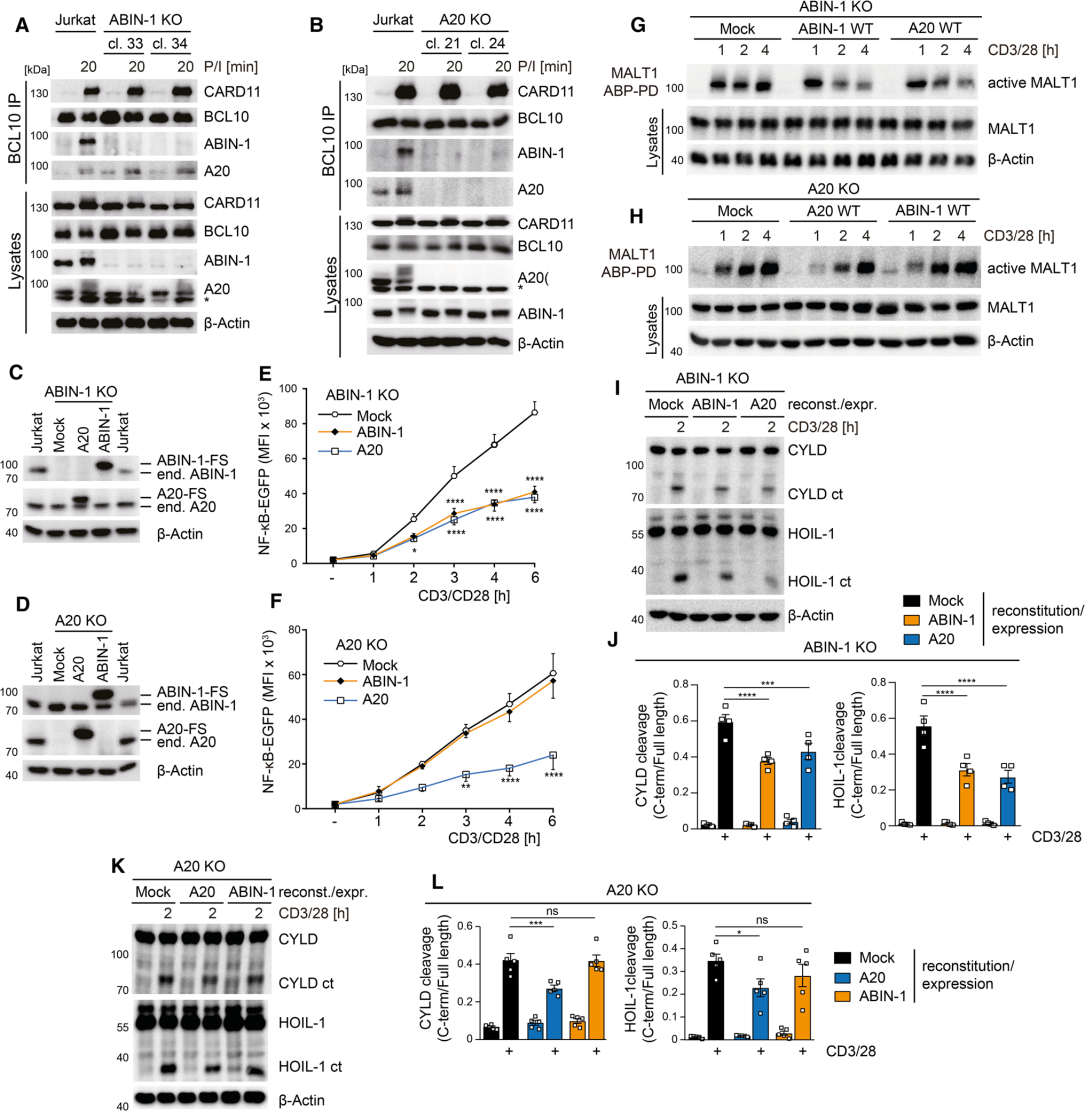
Interdependency of ABIN-1 and A20 in counteracting CBM signaling in Jurkat T cells. **A**, **B** BCL10 IP and detection of BCL10-bound CARD11, ABIN-1 and A20 in parental, ABIN-1 KO and A20 KO Jurkat T cells after P/I stimulation analyzed by Western blot. **C**, **D** ABIN-1 and A20 expression after viral transduction in parental and KO Jurkat T cells analyzed by Western blot. **E**, **F** NF-κB-EGFP reporter activation in transduced ABIN-1 KO or A20 KO Jurkat T cells following CD3/CD28 stimulation (1–6 h) analyzed by flow cytometry. Quantification was done by calculating median fluorescence intensity (MFI). Data represent means ± SEM (*n* = 4) (**E**) or (*n* = 3) (**F**) and quantification was done by two-way ANOVA with Dunnett’s multiple comparisons test, compared with Mock group. *p < 0.05, **p < 0.01, ****p < 0.0001. **G**, **H** Active MALT1 was detected in transduced ABIN-1 KO or A20 KO Jurkat T cells after CD3/CD28 stimulation by MALT1-ABP PD assay and Western blot. **I**, **K** Cleavage of CYLD and HOIL-1 following 2 h stimulation with CD3/CD28 in ABIN-1 KO (**I**) or A20 KO (**K**) Jurkat T cells after viral rescue/expression cells was determined by Western blot. **J**, **L** The ratio of cleaved to full-length CYLD and HOIL-1 was measured by densitometric analyses to quantify the effect of ABIN-1 or A20 reconstitution/expression on MALT1 substrate cleavage shown in I and K. Data represent mean ± SEM (*n* = 4) (**J**) or (*n* = 5) (**L**) and quantification was done by two-way ANOVA with Dunnett’s multiple comparisons test, compared with Mock group. *ns* non-significant, *p < 0.05, ***p < 0.001, ****p < 0.0001

To address a potential crosstalk of A20 and ABIN-1 in counteracting CBM complex functions, we analyzed if ABIN-1 and A20 are recruited to the CBM complex in a co-dependent manner. Both ABIN-1 and A20 associated with BCL10 in Jurkat T cells after P/I or CD3/CD28 stimulation (Fig. 4A,B; Fig. S2A, B). However, while A20 was recruited to BCL10 in ABIN-1 KO cells, ABIN-1 failed to bind to BCL10 in the absence of A20. Thus, ABIN-1 recruitment to the CBM complex is bridged by A20. Neither ABIN-1 nor A20 ablation affected BCL10-CARD11 interaction, indicating intact CBM upstream signaling.

To prove that loss of ABIN-1 and A20 strengthened CBM complex signaling, we reconstituted ABIN-1 and A20 KO Jurkat T cells with respective WT constructs. In addition, to functionally address the putative interdependency of ABIN-1 and A20, we overexpressed ABIN-1 in A20 KO cells and vice versa transduced ABIN-1 KO cells with A20 cDNA. Staining of the co-expressed surface marker ΔCD2 revealed homogenous transduction of all constructs in ABIN-1 or A20 KO Jurkat T cells (Fig. S2C, D). On the protein level, expression of FS-tagged A20 or ABIN-1 was slightly above the endogenous levels, yielding a mild overexpression of the transduced constructs in all settings (Fig. 4C, D). We determined induction of the NF-κB-EGFP reporter gene for 1–6 h after CD3/CD28 stimulation (Fig. 4E, F). Reconstitution of ABIN-1 and A20 effectively reduced the increase of CD3/CD28-induced NF-κB activation in ABIN-1 KO and A20 KO cells, respectively. In addition, A20 overexpression significantly decreased NF-κB activation in ABIN-1 KO Jurkat T cells, suggesting that A20 overexpression bypasses the necessity for ABIN-1 in counteracting NF-κB activation (Fig. 4E). In contrast, ABIN-1 overexpression was unable to interfere with NF-κB activation in A20 KO Jurkat T cells, demonstrating that the negative regulatory function of ABIN-1 strictly relies on the presence of A20 (Fig. 4F).

Next, we assessed the effects of ABIN-1 and A20 reconstitution or overexpression on MALT1 protease activation. MALT1-ABP PD assays revealed that rescue of ABIN-1 or overexpression of A20 impaired CD3/CD28-induced MALT1 protease activity in ABIN-1 KO Jurkat T cells (Fig. 4G). In contrast, only A20 reconstitution, but not ABIN-1 overexpression was able to inhibit MALT1 protease activation in A20 KO Jurkat T cells (Fig. 4H). To test if increased MALT1 protease activity leads to augmented substrate cleavage, we measured CYLD and HOIL-1 in ABIN-1 KO or A20 KO Jurkat T cell after viral reconstitution or overexpression of the two proteins (Fig. 4I, K). We determined the ratio of the cleavage product to the respective full-length protein to quantify the effect of ABIN-1 and A20 on substrate cleavage (Fig. 4J, L). Quantification of the reconstitution experiments confirmed that both A20 and ABIN-1 are mildly counteracting MALT1-dependent substrate cleavage. Moreover, while expression of A20 was able to reduce cleavage of CYLD and HOIL-1 independent of ABIN-1, ABIN-1 was only able to reduce substrate cleavage upon reconstitution, but not in the absence of A20. Thus, while A20 upon overexpression can counteract CBM complex-triggered downstream events independent on ABIN-1, the negative regulatory impact of ABIN-1 relies on the presence of A20.

**Fig. 5:**
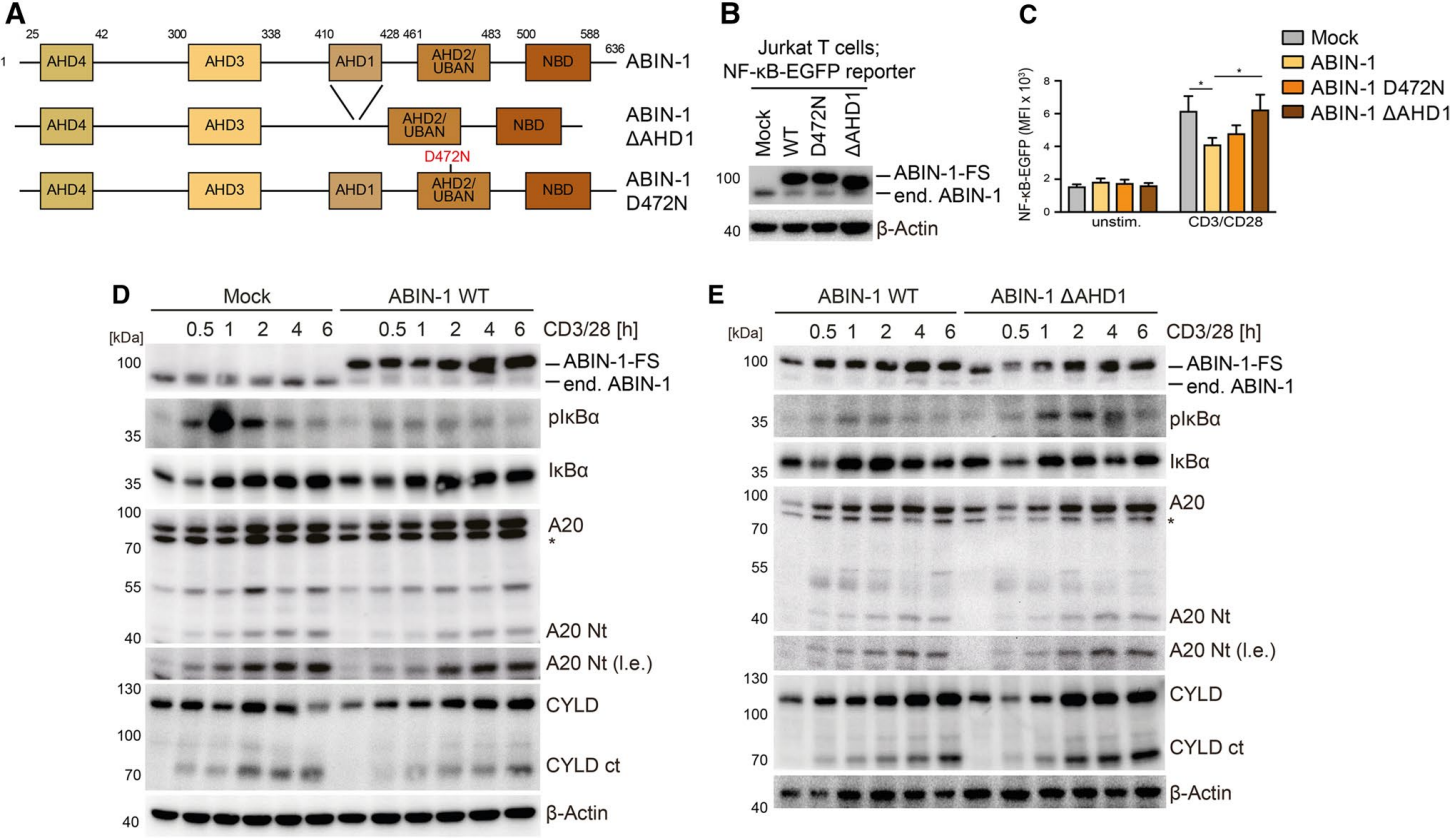
Negative function of ABIN-1 on CBM signaling relies on binding to A20. **A** Schematic depiction of ABIN-1 WT, ΔAHD1 and D472N. **B** Expression of ABIN-1 WT and mt after transduction in Jurkat T cells analyzed by Western blot. **C** NF-κB-EGFP reporter activation in ABIN-1 WT and mt transduced Jurkat T cells following CD3/CD28 stimulation (4 h) analyzed by flow cytometry. Quantification was done by calculating median fluorescence intensity (MFI). Data represent means ± SEM (*n* = 7) and quantification was done by two-way ANOVA with Dunnett’s multiple comparisons test, shown above comparisons. *p < 0.05. **D**, **E** NF-κB signaling and MALT1 substrate cleavage in ABIN-1 WT and ΔAHD1 Jurkat T cells following CD3/CD28 stimulation analyzed by Western blot

To further prove that A20 binding is required for the function of ABIN-1, we compared the effects of ABIN-1 WT, A20-interaction mutant ΔAHD1 (Δaa 410–428) and UBAN-defective mutant D472N in counteracting NF-κB signaling (Fig. 5A) [26, 27]. Deletion of AHD1 in ABIN-1 abolished interaction with A20 (Fig. S2E). Lentiviral transduction led to homogenous infection of Jurkat T cells and robust overexpression of FS-tagged ABIN-1 WT and mutants (Fig. 5B; Fig. S2F). While overexpression of ABIN-1 WT or D472N impaired TCR/CD28-induced NF-κB-EGFP reporter gene induction, ABIN-1 ΔAHD1 was unable to reduce NF-κB activation, underscoring that A20 but not ubiquitin binding is critical for the function of ABIN-1 (Fig. 5C). We used biochemical analyses to demonstrate that ABIN-1 WT counteracted TCR/CD28-triggered IκBα phosphorylation as well as cleavage of the MALT1 substrates A20 and CYLD in Jurkat T cells (Fig. 5D). Direct comparison of ABIN-1 WT and ΔAHD1 mutant revealed that inhibition of NF-κB signaling and MALT1 protease activity required an intact A20 AHD1 domain (Fig. 5E). Thus, the negative regulatory function of ABIN-1 on TCR signaling relies on the ability of ABIN-1 to interact with A20.

### The A20 ubiquitin binding function is required to counteract TCR signaling

**Fig. 6:**
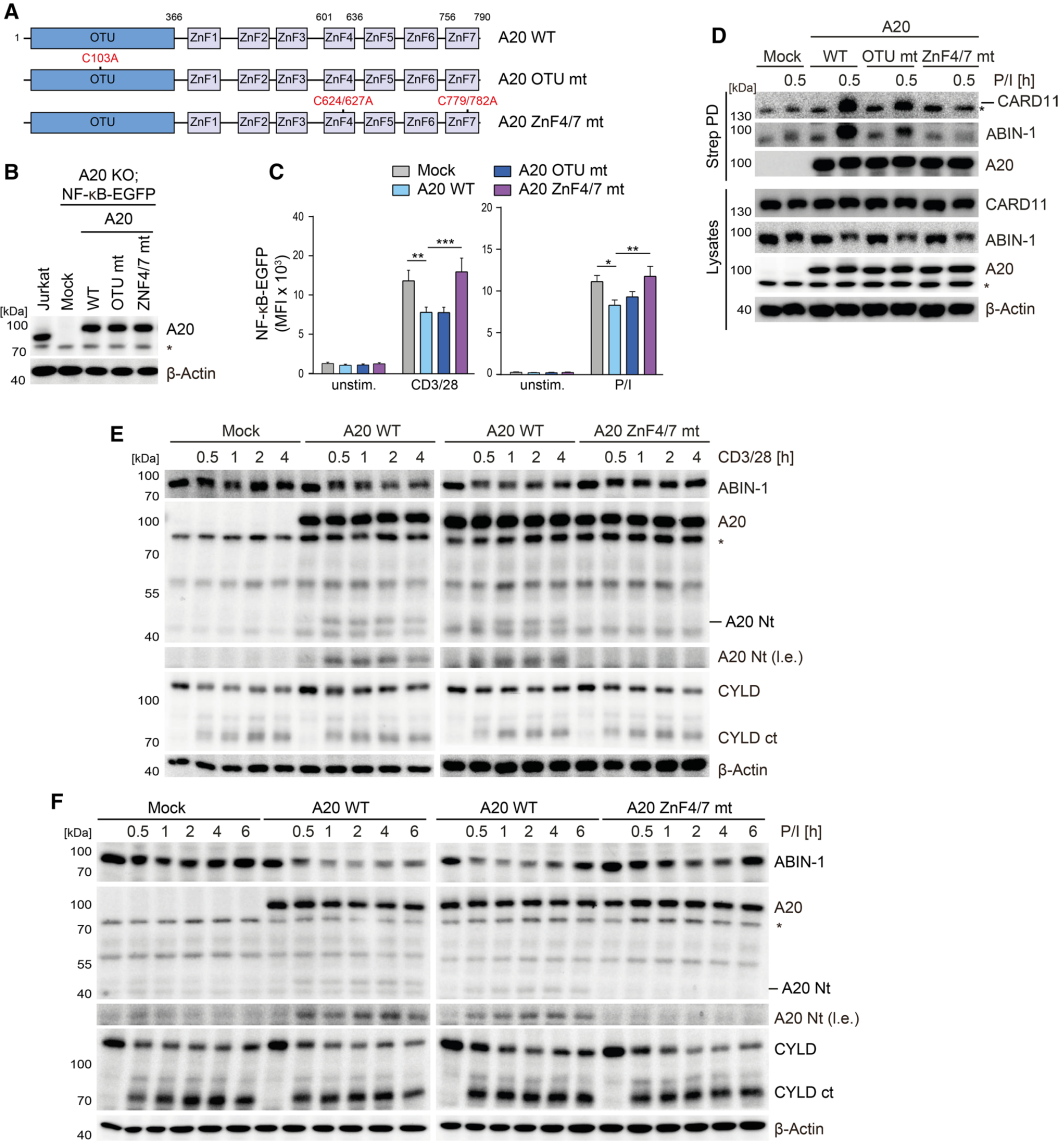
A20 ZnF4 and ZnF7 motifs mediate CBM association and NF-κB suppression in Jurkat T cells. **A** Schematic depiction of A20 WT, OTU and ZnF4/7 mutants. **B** Expression of A20 WT and mt after transduction in A20 KO Jurkat T cells analyzed by Western blot. **C** NF-κB-EGFP reporter activation in A20 KO Jurkat T cells transduced with A20 WT and mt constructs following CD3/CD28 stimulation (4 h) analyzed by flow cytometry. Quantification was done by calculating median fluorescence intensity (MFI). Data represent means ± SEM (*n* = 6) and quantification was done by two-way ANOVA with Dunnett’s multiple comparisons test, shown above comparisons. *p < 0.05, **p < 0.01, ***p < 0.001. **D** Strep-PD and detection of FS-tagged A20-bound CARD11 and ABIN-1 in A20 WT, OTU and ZnF4/7 mutant expressing A20 KO Jurkat T cells after P/I stimulation analyzed by Western blot. **E**, **F** ABIN-1 expression and A20 cleavage analyzed in A20 WT and ZnF4/7 mutant expressing A20 KO Jurkat T cells after CD3/CD28 or P/I stimulation analyzed by Western blot

ABIN-1 is unable to control CBM complex-dependent NF-κB signaling in the absence of A20. Thus, we wanted to investigate, if the N-terminal ubiquitin-hydrolyzing or the C-terminal ubiquitin binding functions of A20 are required for binding to ABIN-1 and silencing of T cell signaling. To this end, we expressed A20 mutant constructs with destructive mutations either in the OTU domain (C103A: OTU mt) or the C-terminal ZnF region (ZnF4/7 mt: C624/627/779/782A) (Fig. 6A) [17]. Lentiviral transduction of NF-κB-EGFP containing A20 KO Jurkat T cells led to homogenous expression of all FS-tagged A20 constructs close to the levels of endogenous A20 in parental Jurkat T cells (Fig. 6B; Fig. S3A). Upon TCR/CD28 or P/I stimulation, A20 WT and OTU mt were impeding NF-κB-EGFP reporter gene induction, while the ZnF4/7 mt prevented the ability of A20 to inhibit NF-κB activation (Fig. 6C). We performed Strep-PD of A20 and found that mutation of ZnF4/7 impaired the ability to interact with CARD11 and ABIN-1 in P/I stimulated Jurkat T cells. Thus, association of A20/ABIN-1 with the assembled CBM complex relies on the C-terminal ubiquitin binding function of A20, which is also necessary to restrict NF-κB activation.

Since A20 ZnF4/7 mutant is not efficiently recruited to the CBM complex, we checked in how far the mutation affects A20 cleavage by the MALT1 paracaspase. While A20 WT or A20 OTU mt proteins were cleaved following CD3/CD28 or P/I stimulation, the A20 ZnF4/7 mt is protected from MALT1-catalyzed cleavage activity (Fig. 6E, F; Fig. S3B, C). In contrast, decline of full-length CYLD is even slightly enhanced in the absence of A20 or in the presence of A20 ZnF4/7 mt, confirming that A20 binding to the CBM complex counteracts MALT1 protease activation (see Fig. 3H) and that the A20 ZnF4/7 mt does not cause a general defect in MALT1 protease function. We tested expression of ABIN-1, which was not severely altered following TCR/CD28 or P/I stimulation in A20 KO Jurkat T cells (Fig. 6E, F). However, reconstitution of A20 caused a steady decline of ABIN-1 in response to TCR/CD28 co-stimulation and a rapid depletion of nearly the entire pool of ABIN-1 after P/I stimulation. Removal of ABIN-1 relied on the intact A20 ZnF4/7 motifs, but did not depend on the catalytic activity by the OTU domain (Fig. 6E, F; Fig. S3B, C), indicating that ubiquitin and ABIN-1 binding to the A20 C-terminal region facilitates ABIN-1 destabilization upon antigenic stimulation of T cells.

### Post-translational crosstalk of A20 and ABIN-1 balances T cell signaling

**Fig. 7:**
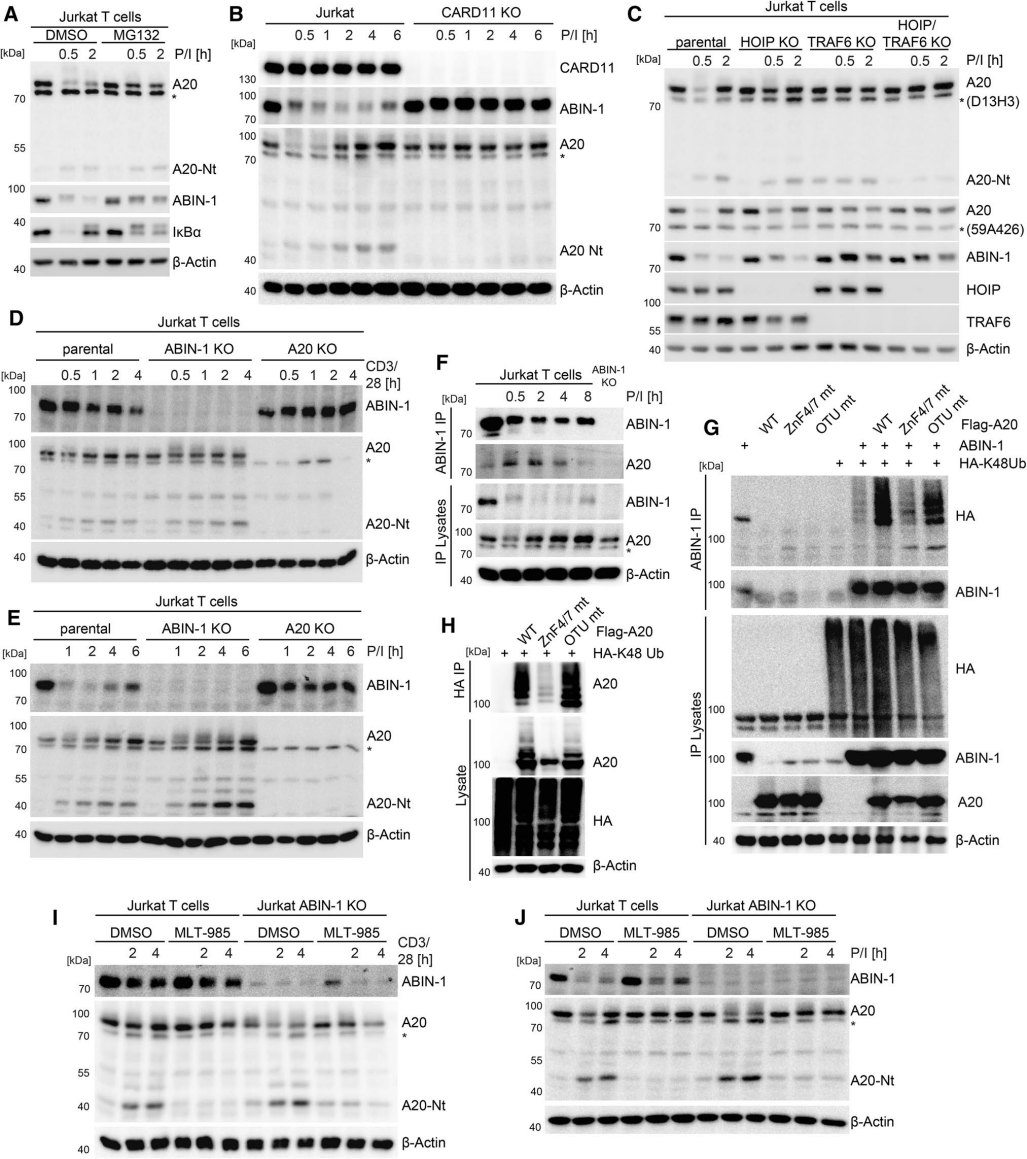
A post-translational crosstalk controls stability of A20 and ABIN-1 after stimulation in Jurkat T cells. **A** Stability of ABIN-1 and A20 in Jurkat T cells after P/I stimulation treated with proteasome inhibitor MG132 (25 µM) analyzed by Western blot. **B**, **C** Stability of ABIN-1 and A20 in parental, CARD11 KO TRAF6 KO, HOIP KO and TRAF6/HOIP double KO Jurkat T cells after P/I stimulation analyzed by Western blot. **D**, **E** Stability of ABIN-1 and A20 in parental, ABIN-1 KO and A20 KO Jurkat T cells after CD3/CD28 and P/I stimulation analyzed by Western blot. **F** ABIN-1-IP and detection of A20-bound ABIN-1 in Jurkat T cells after P/I stimulation analyzed by Western blot. **G** ABIN-1 ubiquitination in HEK293 cells transfected with Flag-A20 WT, ZNF4/7 mt and OTU mt together with ABIN-1 and HA-K48Ub (K48-only) after ABIN-1-IP analyzed by Western blot. **H** A20 ubiquitination in HEK293 cells transfected with Flag-A20 WT, ZNF4/7 mt and OTU mt after HA-IP was analyzed by Western blot. **I**, **J** ABIN-1 stability and A20 cleavage in Jurkat T cells treated with MALT1 inhibitor MLT-985 (1 µM) following CD3/CD28 or P/I stimulation analyzed by Western blot

To understand the crosstalk of A20 and ABIN-1 in balancing T cell activation, we studied the post-translational mechanisms that govern ABIN-1 and A20 expression and protein stability. *ABIN-1* transcripts were not induced upon T cell stimulation or altered in A20-deficient cells (Fig. S4A). As part of a negative feedback loop, NF-κB-dependent transcriptional activation led to *A20*/*TNFAIP3* mRNA induction in response to TCR/CD28 co-ligation, which was further augmented in ABIN-1 KO Jurkat T cells. Similar to IκBα, ABIN-1 degradation following 30 and 60 min of P/I stimulation in Jurkat T cells was impaired by MG132 treatment, demonstrating that both are prone to proteasomal degradation upon T cell stimulation (Fig. 7A). Consistent with previous findings, also the decline in A20 protein was catalyzed by the proteasome [3], while MALT1-catalyzed A20 cleavage in the early phase was unaffected by MG132 treatment. Time-course analyses after TCR/CD28 or P/I stimulation revealed that the prolonged decrease in ABIN-1 and the initial decline in A20 was relying on the proteasomal degradation machinery (Fig. S4B, C). Induction of *TNFAIP3/A20* transcripts prompted re-synthesis of A20 at 2–6 h post stimulation, which was prone to cleavage by MALT1 [3]. Importantly, degradation of ABIN-1 and A20 was abolished in CARD11, BCL10 or MALT1 KO Jurkat T cells, revealing that functional CBM complex formation is required to trigger proteasomal targeting of both negative regulators (Fig. 7B; Fig. S4D, E).

A20 and ABIN-1 are recruited to the CBM complex via the A20 C-terminal ZnF4 and ZnF7 motifs, which bind to M1- and K63-linked ubiquitin chains (see Fig. 6D). Thus, we analyzed if A20 and ABIN-1 degradation is affected by the E3 ligases TRAF6 and LUBAC, which can attach K63- and M1-linked ubiquitin chains to MALT1 and BCL10, respectively [3, 5]. While ablation of LUBAC subunits HOIP or HOIL-1 in Jurkat T cells partially stabilized ABIN-1 and A20, either TRAF6 KO alone or the combined double KO (dKO) of TRAF6 and HOIP nearly abolished ABIN-1 and A20 degradation (Fig. 7C; Fig. S5A–C). In contrast, depletion of cIAP1 by the SMAC-mimetic birinapant did not affect inducible ABIN-1 and A20 degradation, suggesting no involvement of cIAPs (Fig. S5D). Thus, proteasomal degradation of ABIN-1 and A20 relies on CBM complex formation as well as E3 ligase TRAF6 and to a lesser degree the LUBAC, which will facilitate A20 and ABIN-1 recruitment to the CBM complex via conjugation of K63- and M1-linked ubiquitin chains [3, 5].

As noted before, ABIN-1 was stabilized in A20 KO Jurkat T cells after TCR/CD28 or P/I stimulation (Fig. 7D, E). A20 was highly modified and degradation was at least partially impaired in the absence of ABIN-1. Since intact A20 ZnF4/7 motifs are necessary for ABIN-1 degradation (see Fig. 6E, F), we tested if A20 could directly mediate ABIN-1 ubiquitination. Indeed, ABIN-1 and A20 association peaked between 30 min and 4 h of P/I stimulation, which coincided with maximal degradation of ABIN-1 (Fig. 7F). Next, we tested in HEK293 overexpression experiments if ABIN-1 is prone to degradative K48-linked polyubiquitination in an A20-dependent manner. Upon co-expression of ABIN-1 and HA-K48Ub (K48-only chains), A20 promotes the conjugation of K48-linked Ub chains to ABIN-1 (Fig. 7G; Fig. S5E). ABIN-1 ubiquitination relied on the intact ZnF4/7 domains, but not the OTU domain of A20 (Fig. 7G). Further, A20 itself was prone to polyubiquitination upon overexpression in HEK293 cells and conjugation includes K63- or K48-linked ubiquitin chains (Fig. S5F). Interestingly, ubiquitin binding to ZnF4/7 domains but not ubiquitin-hydrolyzing activity by the OTU domain facilitates A20 auto-ubiquitination with degradative K48-linked ubiquitin chains for directing A20 to the proteasomal pathway (Fig. 7H). Thus, proteasomal degradation releases T cells from the negative impact of the A20/ABIN-1 module and K48-linked ubiquitination relies on the C-terminal zinc finger region of A20.

Recruitment to the CBM complex and the TRAF6 E3 ligase, but not ABIN-1 association are necessary for proteasomal A20 degradation in the early phase of T cell stimulation. However, A20 was also prone to MALT1-catalyzed cleavage, which was strongly seen after A20 re-synthesis and augmented in ABIN-1-deficient Jurkat T cells (see Fig. 7D, E). This is in line with our previous observations, showing enhanced and sustained A20 cleavage upon loss of ABIN-1 (see Fig. 3H; Fig. S1F). A20 cleavage was abolished by MALT1 inhibition using the potent allosteric inhibitor MLT-985 or the irreversible inhibitor Z-VRPR-FMK (Fig. 7I, J: Fig. S5G). Importantly, MALT1 inhibition impaired the secondary decrease of full-length A20 protein in ABIN-1 KO Jurkat T cells, showing that enhanced MALT1 protease activity in the absence of ABIN-1 is promoting processing and thus inactivation of A20 after prolonged T cell stimulation. Thus, while A20 triggers proteasomal degradation of ABIN-1 and potentially self-destruction at the initial CBM complex, ABIN-1 in turn counteracts inactivation of re-synthesized A20 by impairing the MALT1-catalyzed cleavage upon sustained CBM complex signaling in activated T cells.

## Discussion

We demonstrate here that the ubiquitin modulators A20 and ABIN-1 cooperate in balancing CBM signalosome activity in T cells. Previous studies have described the negative regulatory function of the ubiquitin-editing enzyme A20 in T cell activation [3, 19]. Our analyses reveal a joint recruitment of A20 and ABIN-1 to the active CBM complex following TCR/CD28 co-stimulation in Jurkat T cells and these findings were corroborated by genetic inactivation in human primary T helper cells. Importantly, while overexpressed A20 can act independent of ABIN-1, the negative impact of ABIN-1 strictly relies on the presence and interaction of A20. Thus, A20 is acting in a dominant manner within the A20/ABIN-1 module, but ABIN-1 exerts its function as an auxiliary factor, which tightly tunes expression, stability and activity of A20. Loss of A20 and ABIN-1 does not lead to chronic T cell activation in the absence of TCR stimulation. In vivo studies showed that T cell-specific A20 deletion enhances T cell effector responses, but does not cause spontaneous T cells activation [9–11]. Thus, A20 and ABIN-1 are responsible for balancing initial and sustained T cell activation post-induction.

**Fig. 8:**
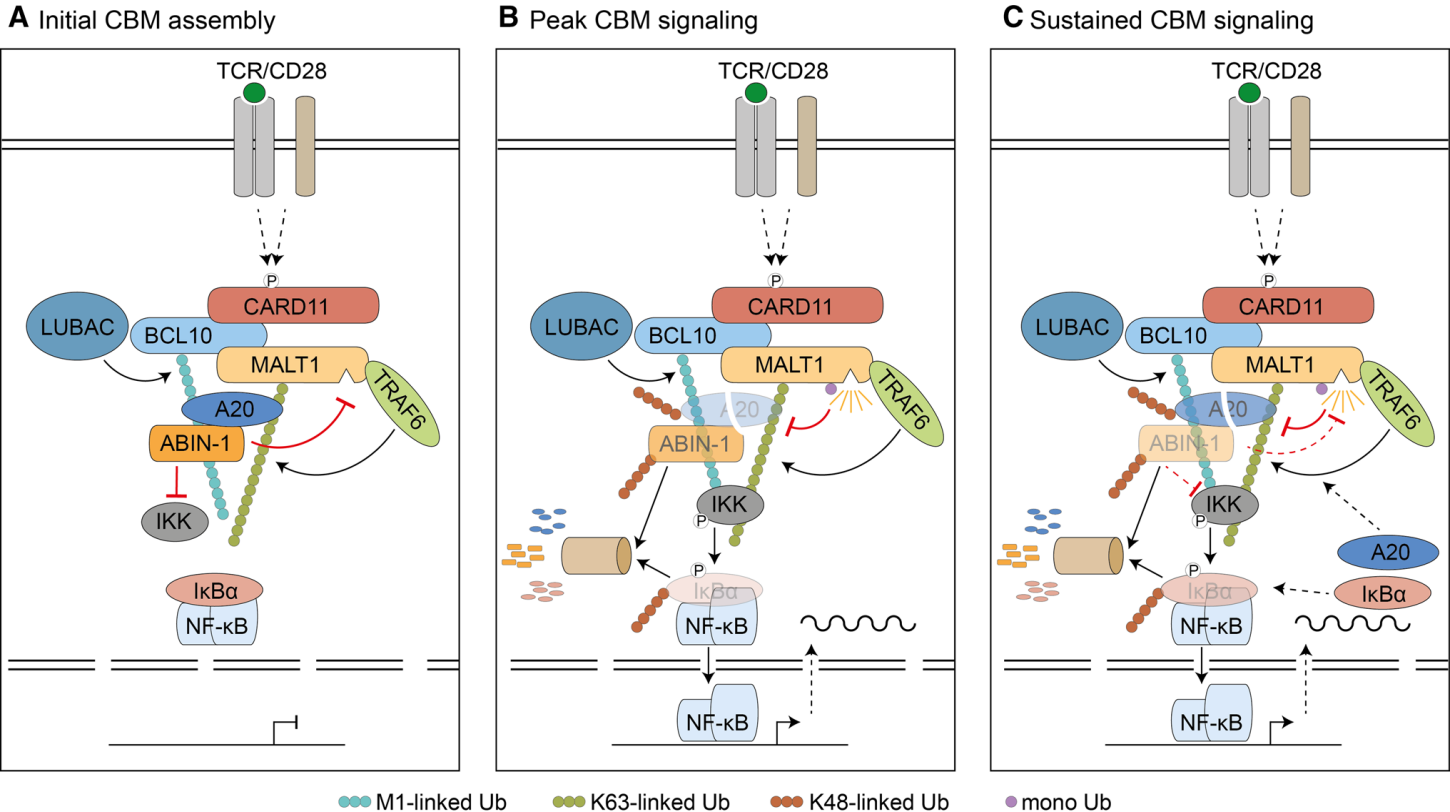
Model for cooperative negative impact of A20 and ABIN-1 on T cell signaling in the initial CBM complex assembly (**A**) in peak CBM signaling (**B**) and sustained CBM response (**C**) after TCR/CD28 co-stimulation

Here, we uncovered an interdependent crosstalk of how A20 and ABIN-1 are controlling post-translational stability of each other, which can help to set thresholds for initial signaling, regulate peak signaling and balance the level of sustained signaling following T cell co-stimulation (Fig. 8). In the first phase, the negative regulators A20 and ABIN-1 are recruited to the initial CBM complex (Fig. 8A). Binding of ABIN-1 is bridged by A20, which associates through its C-terminal ZnF4 and ZnF7 domains with the CBM complex. ZnF4 and ZnF7 serve as binding interfaces for M1- and K63-linked Ub chains, suggesting that A20 and ABIN-1 are linked to the CBM complex via poly-ubiquitin chains conjugated to BCL10 and MALT1 [3, 5, 15, 16, 18]. In line with previous analyses, we find that an intact ABIN-1 UBAN is dispensable for impairing TCR signaling, suggesting that ubiquitin binding of ABIN-1 is not contributing to CBM complex association of A20/ABIN-1 module [27]. In contrast to most cells, which express low amounts of A20 under basal conditions, A20 and ABIN-1 are highly expressed in lymphocytes [50, 51]. Thus, steady-state concentrations of A20 and ABIN-1 may set an initial threshold that limits CBM complex downstream signaling, a mechanism that may protect from undesirable immune activation in response to weak TCR agonists [52].

To foster downstream signaling following CBM complex assembly, ABIN-1 and A20 are removed by the proteasomal degradation machinery, which facilitates IKK/NF-κB and MALT1 protease activation (Fig. 8B). In addition, A20 is cleaved by MALT1 upon recruitment to the CBM complex. Thus, especially the second phase of sustained CBM signaling is characterized by the release from the three negative factors A20, ABIN-1 and IκBα, which allows peak activation of canonical NF-κB signaling. Previous data showed that A20 facilitates K48-polyubiquitination and degradation of RIPK1 after TNFα stimulation or ABIN-1 degradation in response to poly(I:C) stimulation [14, 53]. Here, we show that A20 ZnF4/ZnF7 promotes K48-linked polyubiquitination and degradation of ABIN-1 after TCR stimulation, suggesting that A20 negatively controls ABIN-1 in innate and adaptive immune responses. In addition, ZnF4/ZnF7 facilitates K48-linked auto-ubiquitination and self-destruction of A20. As proposed for RIPK1, other E3 ligases such as Itch or RNF11 may be involved in A20-dependent ABIN-1 or A20 ubiquitination and degradation [54, 55]. Clustering at the CBM complex is obligatory for ABIN-1 and A20 degradation and we demonstrate that the E3 ligase TRAF6 as well as LUBAC components HOIP and HOIL-1 regulate the degradation of the A20/ABIN-1 module. However, the functions of TRAF6 and LUBAC are most likely indirect and restricted to the conjugation of K63- or M1-linked Ub chains to MALT1 and BCL10, respectively [3, 5]. Assembly of these ubiquitin chains is responsible for recruiting A20 via ubiquitin binding ZnF4/7 to the CBM complex, which is a prerequisite for A20 and ABIN-1 degradation. Further, MALT1 protease activity is induced, but maximal substrate cleavage is not yet taking place at early time points and thus cannot account for the rapid A20 decline within ~ 30 min of TCR/CD28 stimulation.

The third phase of sustained CBM signaling is characterized by the NF-κB-induced re-synthesis of the inhibitory molecules IκBα and A20, which would halt IKK upstream and downstream signaling in the absence of ongoing stimulation (Fig. 8C). MALT1 protease exerts a crucial function for sustaining CBM signaling, because MALT1 continually cleaves and inactivates A20, which impairs the negative regulatory effect of A20 on the CBM complex. Thus, MALT1 and A20 are part of an auto-regulatory feedback loop, in which MALT1 cleaves and inactivates A20, which in turn together with ABIN-1 counteracts MALT1 protease activation. Expression of A20 and ABIN-1 is mildly counteracting MALT1 protease activity, which coincides with impaired MALT1 mono-ubiquitination, a critical step for MALT1 protease activation after TCR/CD28 stimulation [48, 56]. Since the ubiquitination machinery controlling MALT1 mono-ubiquitination is unknown, we currently do not know how A20 and ABIN-1 counteract MALT1 protease activity. The A20/ABIN-1 module may either facilitate the recruitment of a DUB that removes mono-ubiquitin or prevent the association of a mono-ubiquitin ligase to MALT1. The A20 ZnF4/7 mutant is not recruited to the CBM complex and resistant to MALT1 substrate cleavage. Compared to other substrates, A20 cleavage is most strongly enhanced in ABIN-1 KO Jurkat T cells. Possibly, ABIN-1 binding to the A20 C-terminus may exert additional effects, such as shielding the MALT1 recognition motif of A20, which could protect A20 from MALT1-catalyzed inactivation. Of note, A20 is highly modified after stimulation in ABIN-1 KO Jurkat T cells, revealing additional layers of crosstalk between these two negative regulators in T cell signaling. Vice versa, ABIN-1 expression is not induced by TCR/CD28 engagement, but ABIN-1 protein amounts are controlled by A20 E3 ligase activity and thus indirectly connected to the regulations that govern A20 expression. Our data reveal that expression and activity of the A20/ABIN-1 module is tightly controlled by an intricate transcriptional and post-translational crosstalk.

We show that the negative regulatory function of A20 on NF-κB signaling in T cells relies on ubiquitin binding through the intact C-terminal ZnF4 and ZnF7 motifs. This is in agreement with the recent findings that underscore the critical function of the A20 ZnF4 and ZnF7 motifs for counteracting pro-inflammatory and innate immune signaling to NF-κB [22, 23]. Mechanistically, we demonstrate that the A20 ZnF4 and ZnF7 motifs are responsible for the association to the CBM complex. Despite the fact that K63-linked ubiquitination on MALT1 is reduced by A20 DUB activity upon overexpression [3], DUB-inactive A20 does not affect NF-κB activation, underscoring that A20 diminishes T cells signaling in a non-catalytic manner. As for MALT1 mono-ubiquitination, it remains unresolved how A20 and ABIN-1 limit MALT1 polyubiquitination. Again, instead of directly hydrolyzing the ubiquitin chains, A20 may utilize other associated DUBs, reminiscent to the necessity of recruiting Itch and RNF11 to foster A20 E3 ligase function [54, 55]. Of note, in vitro the A20 OTU domain preferentially hydrolyzes for K48- over K63-linked ubiquitin multimers [57]. Thus, additional yet unidentified factors may be required for the efficient removal of K63-linked chains from substrates such as RIPK1 or MALT1 [3, 14]. A20 is also recruiting ABIN-1 to the CBM complex, despite the fact that ABIN-1 can interact with K63- and M1-linked ubiquitin chains via the UBAN domain [27]. Disruption of the ABIN-1 UBAN domain did not affect activation of T cells, underscoring that A20/ABIN-1 interaction, but not ABIN-1 ubiquitin binding is critical for antagonizing T cell activation. In sharp contrast, ubiquitin binding by the ABIN-1 UBAN is essential to counteract innate TLR signaling responses [27]. Further, in contrast to the cooperative action that we observe in TCR signaling, A20 and ABIN-1 have independent functions and act synergistic in counteracting TNFα-induced cell death [31]. Thus, depending on the pathways and the cellular context, different mechanisms are governing the counterbalancing function of A20 and ABIN-1 in innate and adaptive immune pathways.

Taken together, we uncovered a complex interplay that determines the expression levels of the ubiquitin modulator A20 and its auxiliary factor ABIN-1 in T cells. Expression and activity of the A20/ABIN-1 module function as a cellular rheostat to tune the strength and the length of CBM complex signaling in T cells.

## Supplementary Information

Below is the link to the electronic supplementary material.

Supplementary file1 (XLSX 1032 KB)

Supplementary file2 (PDF 2270 KB)

## Data Availability

MS raw data on BCL10 protein interactions are deposited to the ProteomeXchange Consortium via the PRIDE [58] partner repository with the dataset PXD028680. The published article includes all other data generated or analyzed during this study.

## Abbreviations

ABIN-1: A20-binding inhibitor of NF-κB-1
AHD: ABIN homology domain
AIP: Aryl hydrocarbon receptor-interacting protein
BCL10: B cell lymphoma/leukemia 10
CARD11: Caspase recruitment domain 11
CBM: CARD11-BCL10-MALT1
CK1α: Casein kinase 1α
CYLD: Cylindromatosis
DUB: Deubiquitinating enzyme
HOIL-1: Heme-oxidized IRP2 ubiquitin ligase 1
HOIP: HOIL-1-interacting protein
IKK: IκB kinase
IP: Immunoprecipitation
LC/MS–MS: Liquid chromatography tandem mass spectrometry
LUBAC: Linear ubiquitin assembly complex
MALT1: Mucosa-associated lymphoid tissue protein 1
NEMO: NF-κB essential modulator
NF-κB: Nuclear factor-κB
OTU: Ovarian tumor
PMA: Phorbol 12-myristate 13-acetate
SHARPIN: SHANK-associated RH domain interactor
TCR: T cell receptor
TLR: Toll-like receptor
TNFα: Tumor necrosis factor α
TNFAIP3: TNFα-induced protein 3
TNIP1: TNFAIP3-interacting protein 1
TRAF6: TNF receptor-associated factor 6
UBAN: Ubiquitin binding in ABIN and NEMO
ZnF: Zinc finger
