# Supplementary material for: A20 and ABIN-1 cooperate in balancing CBM complex-triggered NF-κB signaling in activated T cells
Source: Cell Mol Life Sci. 2022 Jan 31;79(2):112. doi: 10.1007/s00018-022-04154-z (PMC8803816; doi:10.1007/s00018-022-04154-z)
Supplement: Supplementary file 2 — Supplementary file2 (PDF 2270 KB) [file 18_2022_4154_MOESM2_ESM.pdf]

Yin et al.

SUPPLEMENTARY FIGURES AND LEGENDS

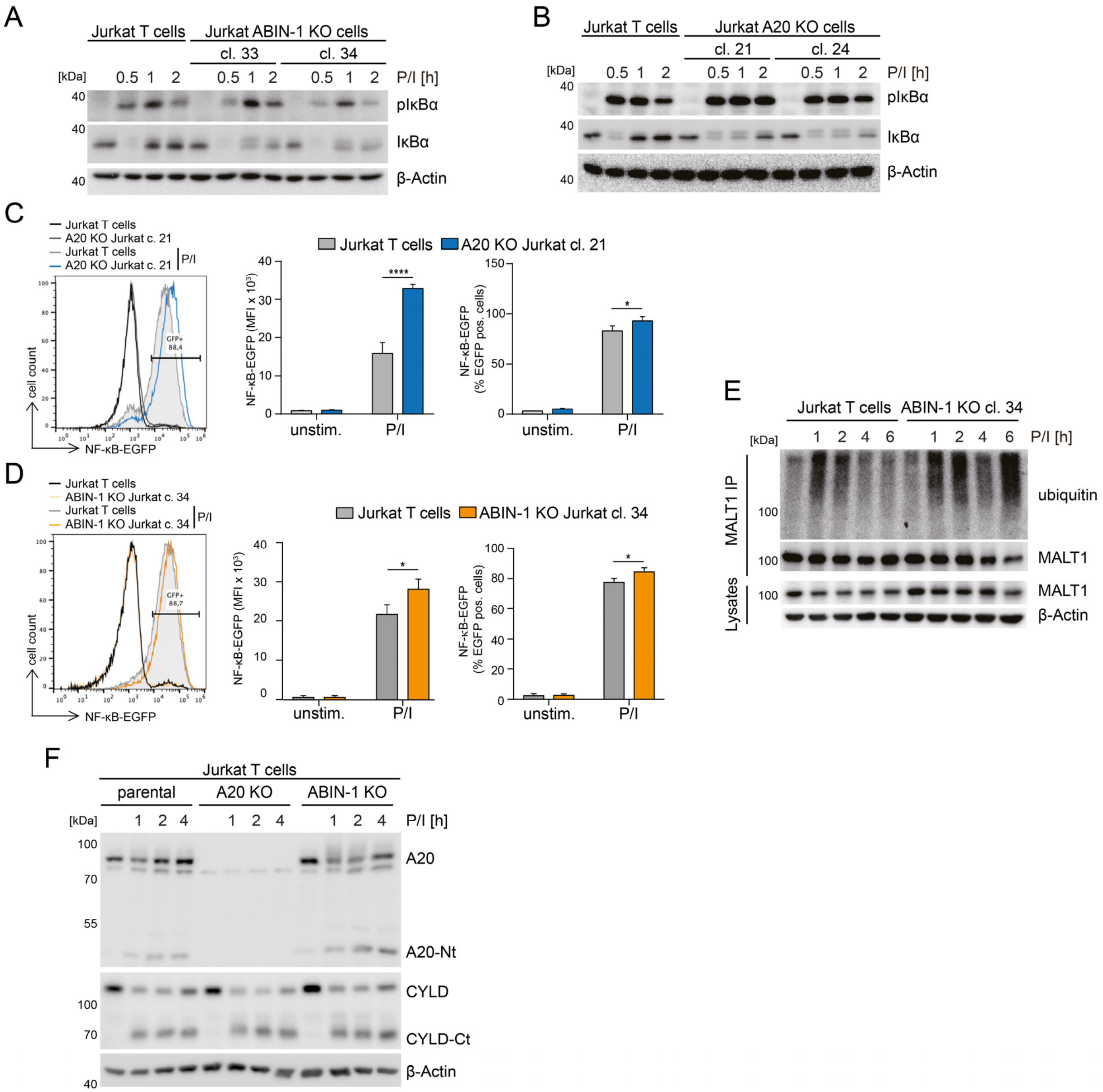

Figure S1: **A, B** NF-κB signaling in parental and KO Jurkat T cells following P/I stimulation analyzed by Western blot. **C**, **D** NF-κB-EGFP reporter activation in parental and KO Jurkat T cells following P/I stimulation (4 h) analyzed by flow cytometry. Quantification was done by calculating median fluorescence intensity (MFI) and number of EGFP positive cells. Data represent means ± SEM (n=3) (C) or (n=5) (D) and quantification was done by two-way ANOVA with Tukey's multiple comparisons test, shown above comparisons. *$p<0.05$, ****$p < 0.0001$. **E** MALT1 polyubiquitination following P/I stimulation. **F** Cleavage of MALT1 substrates in parental, A20 and ABIN-1 KO Jurkat T cells after P/I stimulation analyzed by Western Blot.

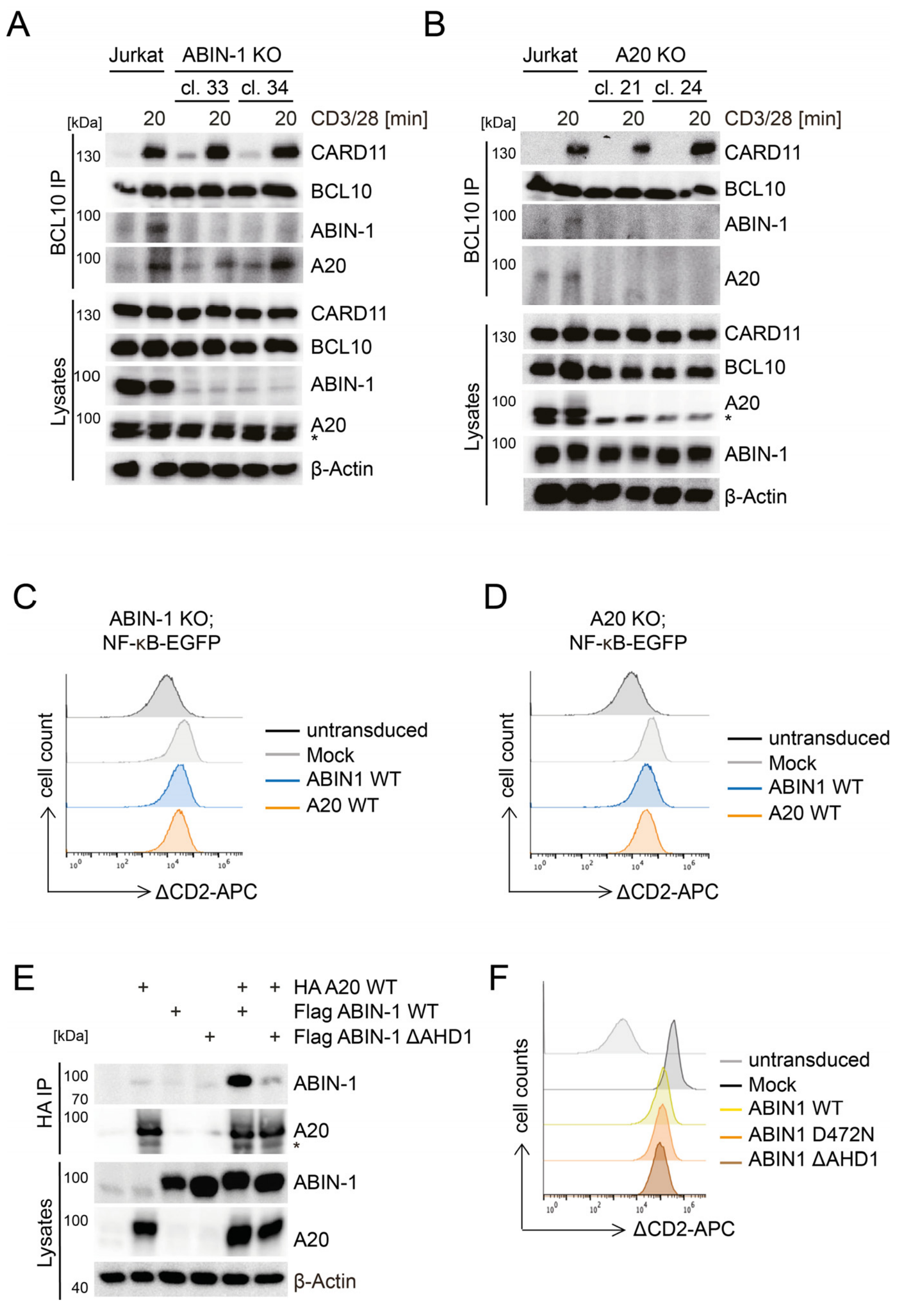

Figure S2: **A, B** BCL10 IP and detection of BCL10-bound CARD11, ABIN-1 and A20 in parental, ABIN-1 KO and A20 KO Jurkat T cells after CD3/CD28 stimulation analyzed by Western blot. **C, D** Transduction of ABIN-1 or A20 in ABIN-1 KO or A20 KO NF-κB-EGFP reporter Jurkat T cells was monitored by the surface-marker hΔCD2 by flow cytometry. **E** HA-A20 binding to Flag-ABIN-1 or Flag ABIN-1 ΔAHD1 mt after transfection in HEK 293 cells and HA-IP analyzed by Western blot. **F** Transduction of ABIN-1 wt and mutants in NF-κB-EGFP reporter Jurkat T cells was monitored by the surface-marker hΔCD2 by flow cytometry.

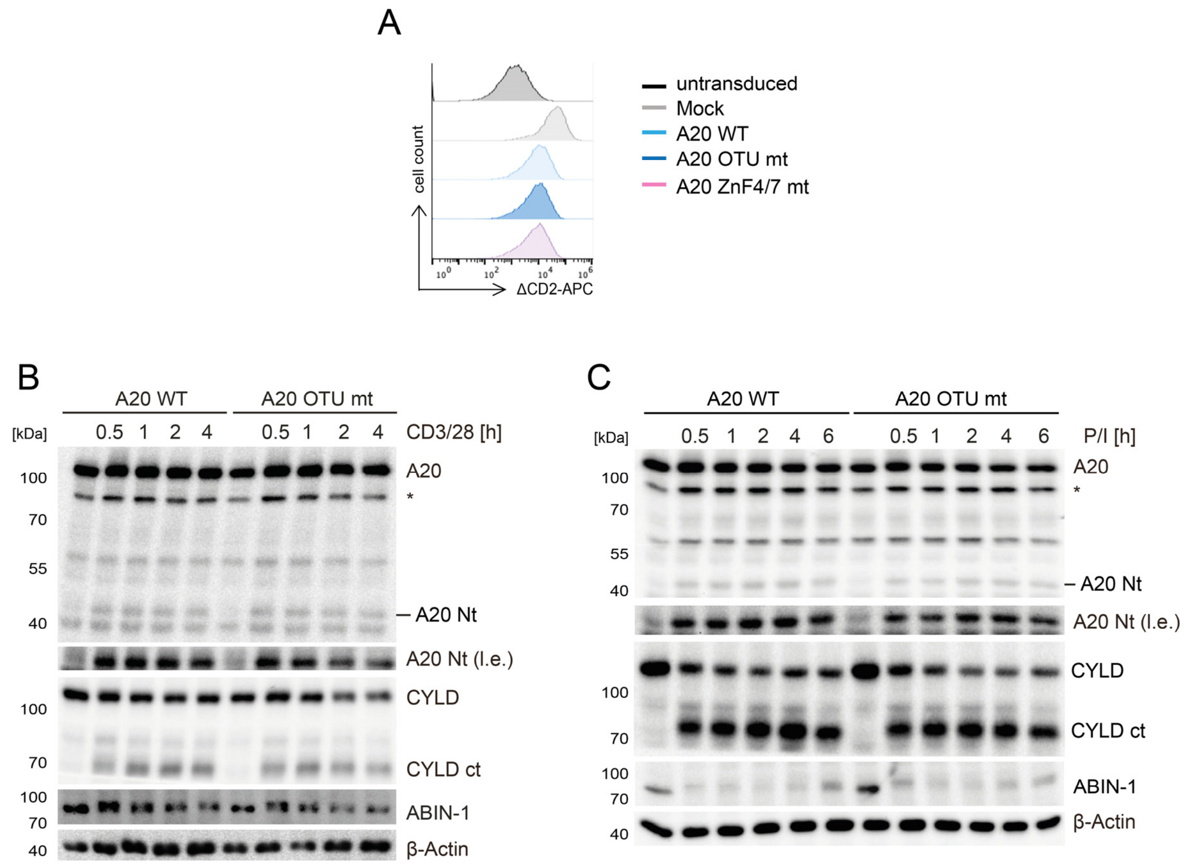

Figure S3: **A** Transduction of A20 wt and mutants in A20 KO NF-κB-EGFP reporter Jurkat T cells was monitored by the surface-marker hΔCD2 by flow cytometry. **B, C** ABIN-1 expression and A20 cleavage analyzed in A20 WT and OTU mutant expressing A20 KO Jurkat T cells after CD3/CD28 or P/I stimulation analyzed by Western blot.

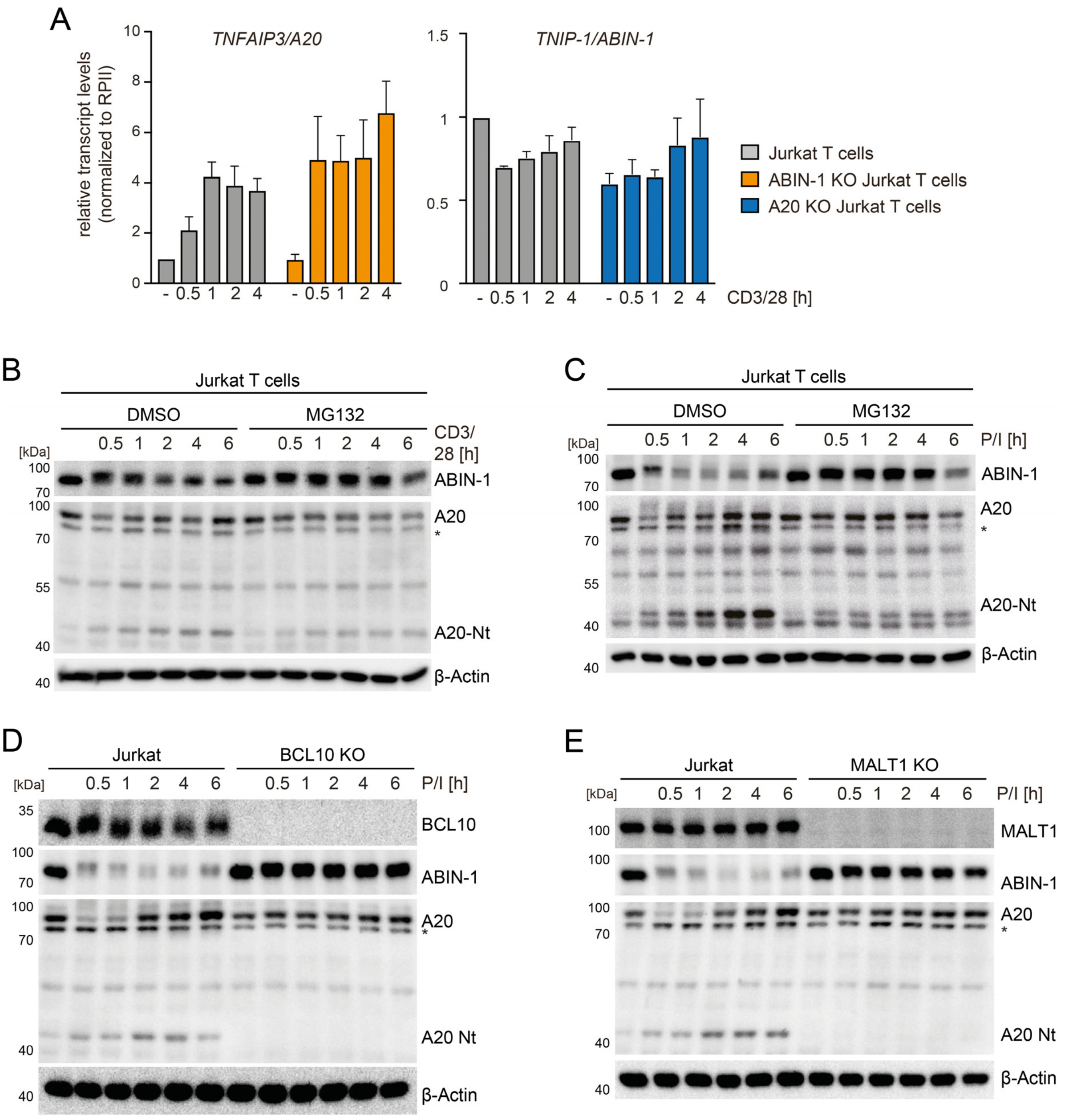

Figure S4: **A** Analyses of mRNA expression induction of *A20/TNFAIP3* and *ABIN-1/TNIP1* in parental and A20 and ABIN-1 KO Jurkat T cells after CD3/CD28 stimulation. Transcript levels were normalized to mRNA levels of *Pol2*. The data represent means ± SEM (n=3). **B, C** Stability of ABIN-1 and A20 in Jurkat T cells after CD3/28 or P/I stimulation treated with proteasome inhibitor MG132 (25 µM) analyzed by Western blot. **D, E** Stability of ABIN-1 and A20 in parental, BCL10 KO and MALT1 KO Jurkat T cells after P/I stimulation analyzed by Western blot.

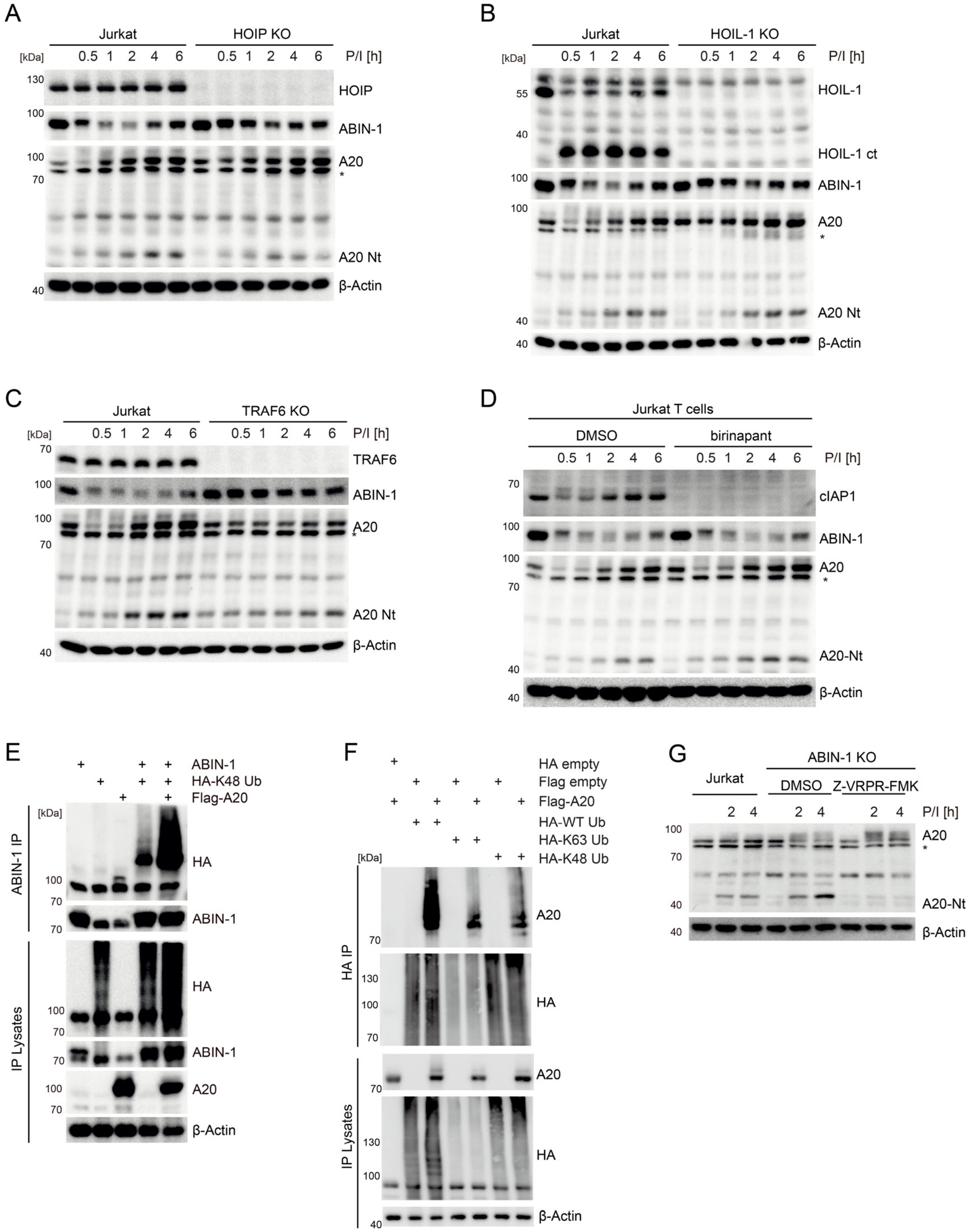

Figure S5: **A-C** Stability of ABIN-1 and A20 in parental, HOIP KO, HOIL-1 KO and TRAF6 KO Jurkat T cells after P/I stimulation analyzed by Western blot. **D** Stability of ABIN-1 and A20 in Jurkat T cell treated with the SMAC mimetic birinapant (2.5 µM) after P/I stimulation analyzed by Western blot. **E** ABIN-1 ubiquitination in HEK293 cells transfected with Flag-A20 WT together with ABIN-1 and HA-K48Ub (K48-only) after ABIN-1-IP analyzed by Western blot. **F** A20 ubiquitination in HEK293 cells transfected with Flag-A20 WT together with HA-WT Ub, HA-K63 Ub (K63-only), HA-K48 Ub (K48-only) after HA-IP analyzed by Western blot. **G** A20 cleavage in Jurkat T cells treated with MALT1 inhibitor Z-VRPR-FMK (75 µM) following P/I stimulation analyzed by Western blot.
